# Drug coated balloons in percutaneous coronary intervention: how can computational modelling help inform evolving clinical practice?

**DOI:** 10.3389/fmedt.2025.1546417

**Published:** 2025-04-30

**Authors:** Silvia Renon, Rafic Ramses, Ankush Aggarwal, Richard Good, Sean McGinty

**Affiliations:** ^1^ Division of Biomedical Engineering, James Watt School of Engineering, University of Glasgow, Glasgow, United Kingdom; ^2^ Glasgow Computational Engineering Centre, University of Glasgow, Glasgow, United Kingdom; ^3^ Fondazione Policlinico Universitario Agostino Gemelli IRCCS, Institute of Cardiology, Catholic University of the Sacred Heart, Rome, Italy; ^4^ Division of Infrastructure & Environment Engineering, James Watt School of Engineering, University of Glasgow, Glasgow, United Kingdom; ^5^ School of Cardiovascular & Metabolic Health, University of Glasgow, Glasgow, United Kingdom; ^6^ West of Scotland Regional Heart & Lung Centre, NHS Golden Jubilee, Glasgow, United Kingdom

**Keywords:** percutaneous coronary intervention, drug coated balloons, drug transport, lesion preparation, computational modelling, *in silico* analysis

## Abstract

Drug-coated balloons (DCB) represent an emerging therapeutic alternative to drug-eluting stents (DES) for the treatment of coronary artery disease (CAD). Among the key advantages of DCB over DES are the absence of a permanent structure in the vessel and the potential for fast and homogeneous drug delivery. While DCB were first introduced for treatment of in-stent restenosis (ISR), their potential wider use in percutaneous coronary intervention (PCI) has recently been explored in several randomized clinical trials, including for treatment of *de novo* lesions. Moreover, new hybrid techniques that combine DES and DCB are being investigated to more effectively tackle complex cases. Despite the growing interest in DCB within the clinical community, the mechanisms of drug exchange and the interactions between the balloon, the polymeric coating and the vessel wall are yet to be fully understood. It is, therefore, perhaps surprising that the number of computational (*in silico*) models developed to study interventions involving these devices is small, especially given the mechanistic understanding that has been gained from computational studies of DES procedures over the last two decades. In this paper, we discuss the current and emerging clinical approaches for DCB use in PCI and review the computational models that have been developed thus far, underlining the potential challenges and opportunities in integrating *in silico* models of DCB into clinical practice.

## 1 Introduction

Cardiovascular disease (CVD) is the largest cause of mortality worldwide, leading to the death of more than 18 million people each year (1). The main cardiovascular pathology is coronary artery disease (CAD), a condition caused by atherosclerosis resulting in partial or complete occlusion of one or more coronary arteries (2–5). As well as contributing to cardiovascular mortality, CAD is the underlying cause of myocardial infarction (MI) and impacts individual quality of life due to symptoms of angina pectoris. To treat MI and angina pectoris patients may undergo percutaneous coronary intervention (PCI), a minimally invasive procedure that inserts tools such as catheters, angioplasty balloons and stents with the final aim of widening the narrowed vessel to restore blood supply. These devices are steered using fluoroscopic guidance to the site of the lesion where they are deployed to widen the lumen and restore blood flow (6, 7).

PCI has evolved since the 1970s from “plain old” balloon angioplasty (POBA) to the adoption of bare metal stents (BMS) before the breakthrough development of drug-eluting stents (DES) (2, 3, 8). Thanks to their ability to elute antiproliferative drug at the lesion site, DES result in a reduction in neo-intimal hyperplasia development, thereby significantly reducing rates of restenosis (re-narrowing). However, in-stent restenosis (ISR) rates for DES are still estimated to be 5%–10% at 1 year (2, 3, 5), with an additional annual target lesion failure rate of 2%–4% beyond one year. These recurrent events affect hundreds of thousands of patients worldwide. Another complication of PCI is stent thrombosis (ST) that may lead to acute vessel closure and MI. However, advancements in DES design mean that less than 1% of patients now suffer from this complication. Despite the low risk of ST, the consequences remain severe with associated mortality rates estimated to be between 20%–40% (5, 9), a window that increases in patients with comorbidities [such as diabetes mellitus, expected to increase globally by more than 50% by 2,045 (2)].

**Figure 1:**
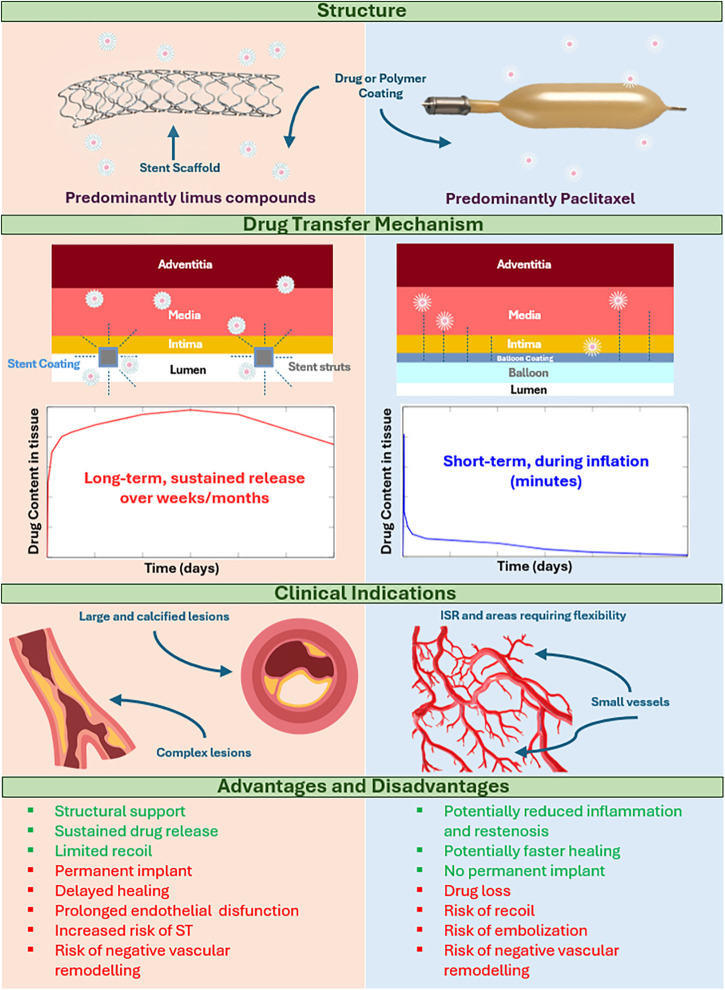
Comparison of drug-coated balloons and drug-eluting stents. *ISR, in stent restenosis; ST, stent thrombosis. This file was partly created using ChatGPT by OpenAI (2025).

Aside from restenosis and ST complications, the lack of biocompatibility and permanency of metallic stents has been associated with delayed healing and prolonged endothelial dysfunction, driving the development of alternative devices that do not leave a permanent scaffold in place. One example is fully bioresorbable scaffolds. These were successfully developed and implanted in the early 2010s but were subsequently withdrawn due to increased rates of scaffold thrombosis compared to 2nd and 3rd generation DES (10, 11). More recently, drug coated balloons (DCB) that combine semi-compliant angioplasty balloon technology with additional drug elution from the surface of the balloon have been gaining in interest and popularity. While DCB were originally developed in the early-mid 2000’s to treat ISR (12–15), features such as high deliverability and lack of a permanent metallic scaffold have seen their use increase in other applications such as small vessel disease (SVD) and bifurcations. Moreover, with the introduction of DCB, high bleeding risk patients who may be excluded from stent treatment due to concerns over prolonged dual anti-platelet therapy (DAPT) could be treated with increased confidence (16–18). With increasing interest in the use of DCB, there has been an evolution in the technology to try to improve drug transfer and retention in tissue. A major advantage of DCB compared to DES is the absence of a permanent prosthesis inside the vessel. Avoiding a permanent metallic implant may reduce the inflammatory response, preserve the original anatomy of the vessel, decrease vascular mismatch and encourage adaptive remodeling (18, 19). However, DCB have limitations including loss of drug during navigation and reduced drug delivery to the endothelial tissues compared to DES (only 10%–20% of the total drug load typically is transferred to vascular tissue (20, 21)). In addition, the absence of a supporting scaffold may increase elastic recoil of the vessel, downstream embolization and, in some cases, induce negative vascular remodeling (19, 22, 23). Unlike DES, drug release from DCB is governed by a short application time (typically between 30 and 120 s), meaning that transport must be fast, efficient and able to penetrate the diseased vascular tissue. A comparison between DES and DCB is provided in Figure 1.

Despite an increase in the use of DCB and development of new DCB technologies by several device manufacturers, the scientific literature on DCB design optimisation is relatively scarce. Indeed, the literature is primarily composed of the clinical evaluation of DCB (late lumen loss, target lesion revascularization, major adverse cardiac and cerebral events, etc.) with few papers focusing on DCB design, interaction with the vessel, drug release and retention. This is in contrast to the extensive literature on these topics with respect to DES (24). Clinical trials to further evaluate the impact of DCB technology are expensive in terms of time and resources. The use of computer model simulation (*in silico* analysis) could reduce the need for more detailed quantitative data for a better understanding of DCB dynamics on a scale that is often beyond clinical measurement resolution (24). A comprehensive analysis of DCB application through computational modelling has the potential to significantly enrich our understanding of these devices, their safety and efficacy and how they, or the PCI procedures in which they are used, may be optimised.

This review article aims to fill a gap in the DCB literature by providing a balanced perspective on both the clinical use of the devices and recent advances in *in silico* modelling. The article starts with an overview of the expanding application of DCB in clinical practice. It then examines the potential of *in silico* models with specific attention to drug release and retention models, structural simulations, and modelling of lesion preparation (LP) devices. Furthermore, a perspective on the potential challenges and opportunities in integrating *in silico* models of DCB into clinical practice is provided

## 2 The use of drug-coated balloons in current clinical practice

While the efficacy and safety of DCB have been established for ISR and native SVD, their potential indications are expanding to all lesions although there is particular interest in bifurcation lesions, large-vessel disease, and high bleeding risk patients (12, 25, 26). Randomized clinical trial data has prompted the International DCB Consensus Group to revise its prior recommendations (25). This updated review not only underscores the established utility of DCB in ISR and SVD but also elucidates their evolving role in diverse clinical scenarios. The collective insights from this comprehensive consensus update serve to inform and guide the contemporary utilization of DCB in coronary artery disease management, thereby advocating for their broader incorporation into clinical practice.

The clinical outcome following the use of DCB vs. DES in CAD is comparable. A systematic review and meta-analysis of 4 studies including a total of 696 patients examined long-term outcomes (>1 year) comparing DCB and DES in the treatment of small vessel CAD and showed that DCB was non-inferior to DES for most outcomes (27, 28), while DCB demonstrated reduced rates of non-fatal MI at 1 year and reduced bleeding rates at 2 years. There was no significant difference in rates of target lesion revascularization (TLR) or target vessel revascularization (TVR) within a follow-up of 9–24 months. However, there was a significant reduction in rates of MI and death in patients receiving DCB compared to those with DES. A small study of 60 patients investigated angiography at 6 months in patients with *de novo* SVD and showed that patients with DES had better immediate angiographically assessed vessel wall expansion, while late lumen loss was significantly less with DCB at 6 months follow-up (29, 30). In another prospective, observational all-comers registry, the safety and efficacy of a DCB-only strategy was assessed in patients with coronary lesions. This registry data suggested DCB could be an attractive alternative to stenting, with high initial success, low major adverse cardiovascular events (MACE) and reduced TLR rates after 9 months (31).

**Figure 2:**
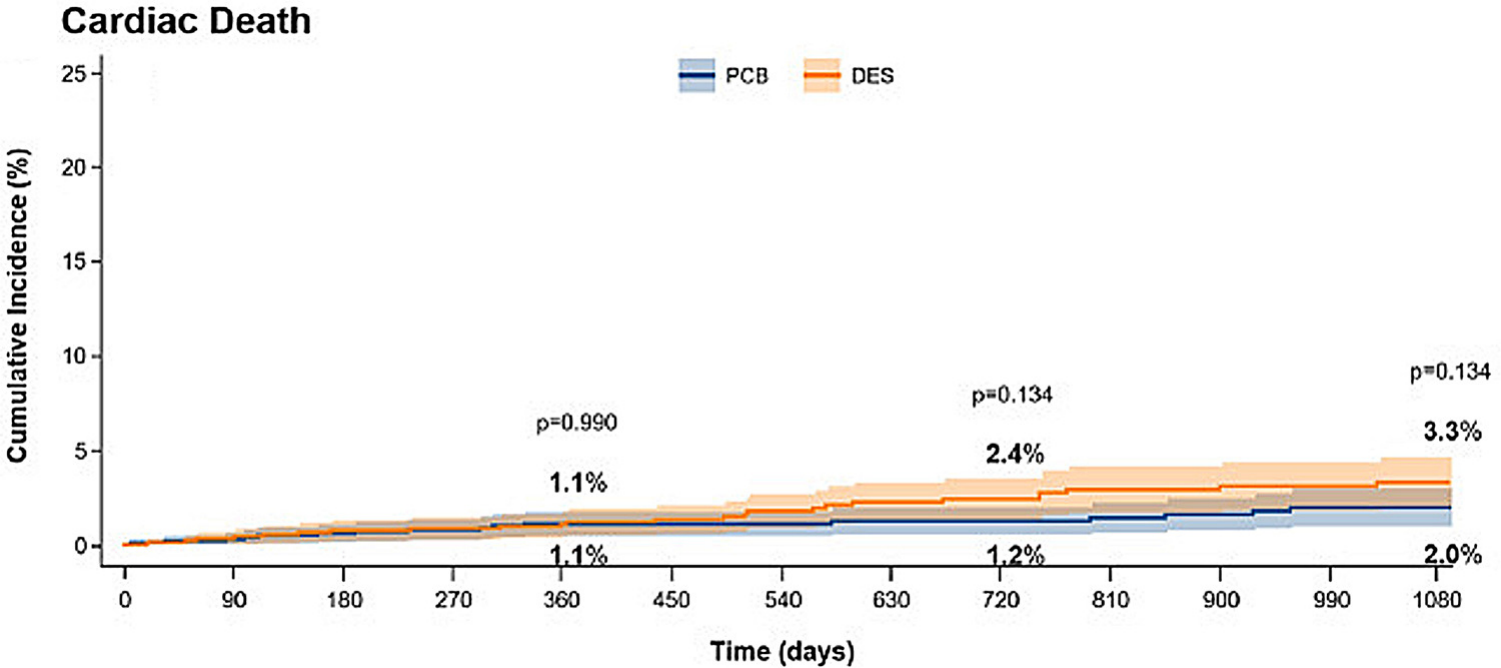
Incidence of death in patients treated for ISR with paclitaxel coated balloons (PCB) or paclitaxel-DES. Obtained from (25) under the terms of the Creative Commons Attribution Non-Commercial License (http://creative commons.org/licenses/by-nc/4.0/).

To date, the majority of clinical trials have assessed the safety and efficacy of paclitaxel-coated DCB. Paclitaxel is a lipophilic, rapidly absorbed anti-proliferative drug that has prolonged tissue retention and was the first drug used for DES coatings. However, new generation stents almost exclusively use sirolimus or its analogues. These antiproliferative agents have proven to be superior to paclitaxel to reduce restenosis and cardiovascular events following DES implantation (25, 32). However, the use of limus drugs on DCB is more challenging as these drugs are less lipophilic than paclitaxel and therefore their tissue penetration and retention is reduced. Innovative technologies have been sought to improve the delivery and retention of sirolimus in tissue following DCB deployment, including the use of dedicated polymers to create micro-reservoirs. Thanks to these developments, the use of limus DCB has become more prominent in ISR cases (33) and emerging studies are now challenging DES implantation as the definitive strategy in *de novo* coronary lesions (34) (Figure 2). Therefore, further comparative studies between paclitaxel and limus DCB are required (35).

### 2.1 *De novo* lesions

In the treatment of *de novo* coronary lesions, initially a DCB angioplasty strategy followed by bare metal stent (BMS) implantation was used, aiming to combine the antiproliferative effect of the DCB with the mechanical properties of the metallic stent (36–38). However, this approach yielded inferior outcomes compared to DES-only strategies, prompting a shift towards a DCB-only approach. The concept of DCB-only PCI involves treating coronary stenosis with minimal reliance on permanent or semi-permanent implants, predominantly reserving them for lesions at heightened risk of acute vessel closure or unfavorable long-term outcomes. Studies have demonstrated the efficacy of DCB compared to both BMS and DES in *de novo* lesions, particularly in challenging anatomies of SVD and bifurcation lesions (39, 40). However, DCB have also shown potential as an alternative to DES for a wide range of lesion types, offering comparable mortality rates (cardiovascular and all-cause), with additional benefits in terms of TLR and shorter DAPT duration (39, 41). This may be particularly relevant for high bleeding risk patients who do not tolerate prolonged antiplatelet therapy (36, 41). Numerous clinical trials have been performed to test DCB use in *de novo* lesions within a complex scenario Table 1.

**Table 1:** Clinical trials of DCB only vs. DES in *de novo* coronary lesions.

Study	Lesion type	Comparators to DCB and combinations	n patients (DCB vs. DES)	Follow-up duration	Angiographic follow-up	p Value	MACE (%)	p Value	TLR (%)	p Value
PICCOLETTO	Small vessel disease	PCB vs. PES	60 (29 vs. 31)	1,6 and 9 (clinical) 6–8 months (angio)	DS (%): 43.6 ± 27.4 vs. 24.3 ± 25.1	0.029	35.7 vs. 13.8	0.054	32.1 vs. 10.3	0.15
BELLO	Small vessel disease	PCB vs. PES	181 (90 vs. 91)	1 and 6 months (clinical) 6 months (angio)	LLL (mm): 0.08 ± 0.38 vs. 0.29 ± 0.44 BR (%): 10 vs. 12.4 DS (%): 32.31 ± 16.66 vs. 26.69 ± 20.38	0.001 0.64 0.06	10 vs. 16.3	0.21	4.4 vs. 7.6	0.37
BELLO follow-up	Small vessel disease	PCB vs. PES	181 (90 vs. 91)	2 years (clinical)	N/A	N/A	14.4 vs. 25.3	0.08	6.8 vs. 12.1	0.23
BASKET SMALL 2	Small vessel disease	PCB vs. (PES+EES)	758 (382 vs. 376)	6 and 12 months (clinical) 6 months (angio)	LLL (mm): 0.13 (−0.14 to 0.57) vs. 0.10 (−0.16 to 0.34)	0.72	7.5 vs. 7.3	0.918	3.4 vs. 4.5 (TVR)	0.4375
BASKET SMALL 2 Follow-up	Small vessel disease	PCB vs. (PES+EES)	758 (382 vs. 376)	3 years (clinical)	N/A	N/A	15 vs. 15	N/A	9 vs. 9 (TVR)	N/A
RESTORE SVD	Small vessel disease	PCB vs. ZES	230 (116 vs. 114)	1, 6 and 12 months (clinical) 5 years (clinical) 9–12 months (angio)	LLL (mm): 0.26 ±0.42 vs. 0.30 ± 0.35 BR (%): 11 vs. 7 DS (%): 29.3 ± 20.2 vs. 22.8 ± 15.3	0.41 0.01 0.40	9.6 vs. 9.6	1.00	4.4 vs. 2.6	0.72
BIOLUX-I	Bifurcations	MB: EES vs. SB: PCB	35	1, 6, 9 months (clinical) 1 year (clinical) 9 months (angio)	LLL (mm): −0.03 ± 0.22 vs. 0.01±0.12 BR (%): 0 vs. 0 DS (%): 22.6 ± 12.2 vs. 12.4 ± 9.2	N/A	5.7 (PBC)	N/A	2.9	N/A
DEBSIDE	Bifurcations	MB: EES vs. SB: PCB	50	1, 6 and 12 months (clinical) 6 months (angio)	LLL (mm): −0.04 ± 0.34 vs. 0.69 ± 0.46 BR (%): 2.0 vs. 14.0 DS (%): 25.7 ± 12.6 vs. 31.9 ± 18.8	N/A	10 (PBC)	N/A	6	N/A
BEYOND	Bifurcations	MB: DES vs. SB: PCB or BA	222 (DCB 113 vs. BA 109)	9 months (clinical) 9 months (angio)	MB LLL (mm): 0.12 vs. 0.08 SB LLL (mm): −0.06 ± 0.32 vs. 0.18 ± 0.34	0.72 < 0.0001	0.9 vs. 3.7	0.16	0 vs. 0	N/A
Costopoulus et al.	Diffuse disease	PCB+DES vs. DES alone	69	2 year (clinical) After procedure (angio)	DS (%): 26.5 ± 7.93 vs. 15.6 ± 4.98	<0.01	20.8 ± 6.1 vs. 22.7 ± 4.5	0.71	14.8 ± 5.7 vs. 11.5 ± 3.4	0.44
Yang et al.	Diffuse disease	PCB only and PCB+DES vs. DES only	1027 (355 DCB vs. 672 DES)	3, 6, 9, 12 months (clinical) 3 years(clinical) 9–12 months (angio)	LLL (mm): 0.06 ± 0.61 vs. 0.41 ± 0.64 DS (%): 31.96 ± 17.21 vs. 30.67 ± 18.80	0.622 < 0.001	11.0 vs. 13.7	0.324	8.7 vs. 9.8	0.652

DCB, drug coated balloons; PBC, paclitaxel coated balloons; PES, paclitaxel eluting stents; EES, everolimus eluting stents; ZES, zotarolimus eluting stents; BA, balloon angioplasty; DES, drug eluting stents; angio, angiographic; DS, diameter stenosis; LLL, late lumen loss; BR, binary restenosis; MB, main branch; SB, side branch; MACE, major adverse cardiac events; TLR, target lesion revascularization; TVR, target vessel revascularisation; N/A, non applicable.

### 2.2 Complex lesions

Complex coronary lesions pose significant challenges for the interventional cardiology community, particularly in patients with a higher bleeding risk. Diffuse atheroma, bifurcations and chronic total occlusion (CTO) lesions constitute around 20% of all PCI cases (42) and are often associated with suboptimal outcomes when managed with traditional therapeutic strategies. However, the advent of DCB angioplasty, both as a standalone procedure or in conjunction with other devices, offers new hope for better patient outcomes by tailoring treatment strategies to the unique demands of these complex scenarios. In current practice, treatment of complex coronary disease often involves placement of long, or overlapping stents within the lesion. However, stent length and the use of 2-stent bifurcation techniques have been identified as independent predictors for future events including ISR and ST (43). Overlapping stents, especially those exceeding 60 mm in length, have been associated with a higher rate of target lesion revascularization (TLR) (44, 45).

#### 2.2.1 Diffuse disease and long lesions

Recognising the increased risks of target-vessel failure (TVF) with long areas of overlapping DES and in smaller calibre vessel, hybrid methods that combines DES and DCB applications to minimize stent length have been proposed to treat diffuse disease, particularly where the calibre of the vessel changes. Usually the strategy involves DES implantation in the larger, more proximal segments of the diseased vessel, with DCB utilized in the smaller, more distal part (45, 46). A similar approach was used for long lesions were the hybrid approach consisted of DCB deployment in the distal portion of the vessel and DES implantation in the proximal segment of the vessel (47). DCB angioplasty requires pre-dilatation of the stenosed segment to ensure effective outcomes. These studies used residual stenosis of less than 50% after preparation balloon dilatation as a cut-off for final DCB angioplasty with bail-out stenting reserved for cases where there was residual stenosis greater than 50% after predilatation (42).

#### 2.2.2 Bifurcation lesions

Bifurcation lesions, with their patient-specific anatomical challenges and inherent complexity, represent approximately 15% of PCI cases (48, 49). Historically, left main coronary artery (LMCA) bifurcation lesions were treated with coronary artery bypass graft surgery (CABG). However, a significant proportion of patients are not suitable for CABG and advances in PCI have paved the way for DES to become a viable alternative for some low-risk patients. Where appropriate, bifurcation-specific techniques including the kissing balloon technique, T-stenting and protrusion (TAP), culotte and double kissing (DK) crush techniques may be deployed (50–52). However, the dynamic landscape continues to evolve, and recent studies have highlighted the safety and efficacy of DCB as an option (53). This has sparked interest in novel PCI strategies that might prioritize a “less is more” approach, focusing on reducing stent implantation using hybrid techniques that might soon set new standards for bifurcation lesions (47). In the BIOLUX-I Study (48) a strategy of pre-dilation with a DCB in the main branch prior to BMS implantation was inferior to the combination use of DES and uncoated balloon for left main stem intervention. In the DEBSIDE study (54) of bifurcation intervention, main vessel and side branch dilatation was performed sequentially, followed by DES (Nile PAX stent) deployment in the main branch and DCB (DANUBIO balloon) inflation in the side branch. In the BEYOND study (49) a paclitaxel eluting stent was deployed in the main branch followed by a kissing balloon inflation with regular balloons and finally DCB inflated in the side branch. These various approaches demonstrate complexity of bifurcation lesions and the strategies that may be used to treat these lesions. While certain techniques focus on combining DES and DCB, others explore the most effective order of treatment.

#### 2.2.3 Small vessel disease

Small vessel disease (SVD) treatment in CAD constitutes a challenge to maintain vessel patency and performance efficacy following PCI. The use of DES in SVD cases is associated with higher ISR and ST rates. The introduction of DCB technology has heralded a new approach for SVD. Several clinical trials have compared DES vs. DCB use for SVD in the coronary arteries with numerous trials seeking to establish superiority of either technique, with contradicting results. In the PICCOLETTO trial (55) DCB failed to prove equivalent performance to DES for angiographic endpoints in SVD. However, it may be that this result was due to reduced efficacy of the DCB (Dior, Eurocor) used, that was later replaced by a newer generation. In addition, suboptimal outcomes for DCB may have been caused by inadequate LP, e.g., pre-dilatation was only performed in 25% of cases compared to 96.8% of cases in BELLO trial. In the latter study, paclitaxel DCB was associated with less angiographic late lumen loss and comparable restenosis and revascularization rates to paclitaxel eluting stents (56, 57).

In the open-label, randomized BASKET SMALL 2 trial (58–62), the non-inferiority of DCB vs. DES was tested. DCB was non-inferior to DES for MACE at 12 month follow-up. These results were comparable with the RESTORE SVD trial (63) that tested SVD and very small vessel disease (VSVD) applications. The RESTORE DCB was non-inferior to the RESOLUTE DES while DCB and DES had comparable 1-year rates of target lesion revascularization. In a study dedicated to acute myocardial infarction (AMI) in SVD, DCB had comparable results with DES in terms of major adverse cardiovascular events (MACE) with reduced ST. For this reason, it was concluded that DCB can be adopted as a valid substitute to DES for the treatment of *de novo* lesions causing AMI. There are still limited data to support the wider application of DCB for the treatment of AMI caused by *de novo* lesions, in particular, the absence of long-term follow up (64). More recently, a systematic review and meta-analysis compared angiographic and clinical outcomes of DCB vs. DES in SVD (51). It was observed that DCB are associated with less late lumen loss (LLL) and MI while DCB and DES were comparable in terms of MACE, all-cause death, TLR and target vessel revascularization (TVR), as depicted in Table 2. The complex and narrow geometry of small coronary vessels, may explain higher rates of restenosis following implantation of small calibre DES compared with DCB.

**Table 2:** DCB vs. DES meta-analysis outcomes in small vessel coronary artery disease (65).

Failure cause	DES %	DCB %
Periprocedural myocardial infarction	3.91%	2.24%
Myocardial infarction	3.31%	1.55%
Major adverse cardiovascular events	10.22%	8.72%
All-cause mortality	2.38%	1.17%
Target lesion revascularization	7.14%	7.92%
Target vessel revascularization	7.83%	8.15%

### 2.3 Lesion preparation techniques and clinical approaches

Lesion preparation (LP) is a crucial step in all PCI procedures. It entails pretreatment of the lesion by means of targeted tools to dilate the occluded vessel, modify lesion characteristics and facilitate either stent or balloon insertion and expansion at the site. It has been proven that adequate LP systematically applied prior to either DES or DCB insertion improves outcome including reduced rates of ISR, TLR and MACE (66, 67). LP is now standard practice in the majority of CAD interventions and is the first lesion modifying step in most PCI procedures (68). LP is even more relevant when it comes to DCB application (69). In cases of DCB-only procedure it has been proposed that a target value of residual stenosis be regarded as adequate LP to predict efficacy. This value should be enough to minimise residual flow limiting stenosis and prevent recoil whilst avoiding the risk of dissection that may require bail out stenting (% of plaque area <58.5%) (70). Current DCB studies in *de novo* CAD suggest a residual stenosis <30% be regarded as adequate LP (71). Evidence supports improved outcomes with good LP, while DCB applications without pretreatment of the lesion caused either negative outcomes or procedure failure (72–74).

Different techniques for LP can be used based on the complexity of the lesion, e.g., it’s composition, position, and morphology. For uncomplicated lesions, guidelines recommend using a semi- or non-compliant angioplasty balloon with a diameter equal to the diameter of the vessel (1:1 ratio). When balloon delivery is challenging due to complexity or stenosis severity, small caliber balloons and vasodilators can be used initially. If semi-compliant balloon expansion fails at nominal inflation pressures, then high-pressure non-compliant balloon dilatation or the use of modifying balloons are suggested. Modifying balloons can be categorized as either cutting balloons or scoring balloons. The former is a rigid non-compliant balloon with sharp blades adherent to the surface of the balloon (atherotomes) in a longitudinal orientation (75). Cutting balloons are designed to create fissures in either fibrous or calcified atheroma to weaken this tissue and facilitate expansion. However, they can be challenging to maneuver through tortuous vessels and carry an increased risk of perforation. The scoring balloon (76) has wires adherent to the outer balloon surface, again orientated longitudinally. During balloon inflation, plaque disruption is achieved through pressure application and localized force transmission from the wires. Scoring balloons have better deliverability compared to cutting balloons but still less than angioplasty balloons and can be challenging to use in tortuous, calcified anatomy (77).

Whenever balloon angioplasty LP does not produce adequate results, other techniques might be adopted, such as high speed rotational atherectomy (rotablation), laser atherectomy orbital atherectomy, lithotripsy or excimer laser coronary angioplasty (ELCA) (78, 79). These techniques are generally used to treat severely stenotic plaques or plaques that are highly resistant to dilatation. Usually, the underlying pathology is extensive calcification within the intimal layer of the vessel wall [comprising 30% of all coronary lesions (77)]. Excessive calcification is the primary cause of balloon and stent under expansion and can be particularly troublesome in bifurcation lesions. These adjuvant devices disrupt and fracture calcium through the delivery of either electrical or mechanical energy. Atherectomy, either rotational atherectomy (RA) or orbital atherectomy system (OAS), is based on the use of a high-speed rotating burr with a head coated with diamond chips that modifies plaque by selectively engaging with hard calcified tissue. Soft tissue is deflected away and does not interact with the burr whilst hard, calcified tissue is disrupted. RA is a challenging procedure, requiring training and experience and is associated with increased adverse complications. Intravascular lithotripsy (IVL) uses sonic pulses to selectively fracture calcified plaques. It has better crossability than cutting balloons, requires less training than atherectomy and aims to reducing trauma produced by blades and burrs (80). Some particularly calcified lesions may require further intervention and a new hybrid therapeutic approach has been designed: the RotaShock, which combines the use of RA and a Shockwave IVL balloon. This method has proved to be effective and safe although the major drawback is related to the high cost of the procedure (81). The use of these complex adjuvant lesion modifying techniques prior to DCB use is an area of increasing interest.

#### 2.3.1 Optimal LP for DCB only strategy

The aim of LP is to facilitate optimal balloon angioplasty or DES implantation, and optimise to facilitate drug transport. Evaluation of LP is usually carried out by assessment of balloon expansion following preparation, aiming for 1:1 balloon:vessel expansion. This assessment is performed before a final decision on DCB or DES use.

**Figure 3:**
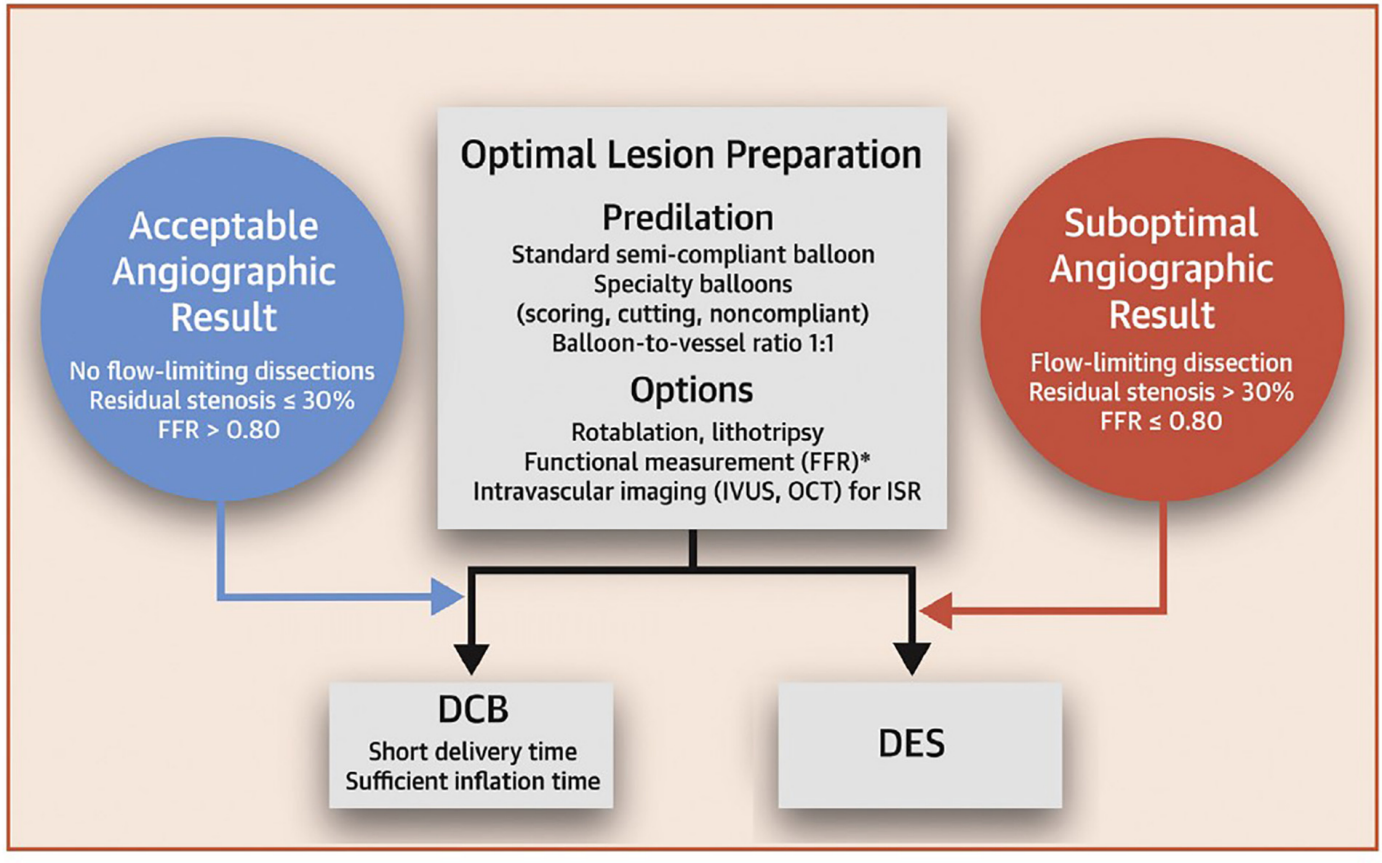
DCB-only strategy workflow for PCI. Obtained from (66) under the Creative Commons CC-BY-NC-ND licence.

Based on consensus guidelines (66), if successful balloon expansion is accomplished with recoil <30%, no residual flow limiting stenosis and no significant dissection, it is recommended that DCB may be used to treat the lesion. However, where these criteria are not met, the guidelines propose using a DES approach. An ideal LP is attained when the inflated balloon fully expands to the appropriate size for the treated vessel while the residual stenosis following balloon withdrawal is below 30%, there are no flow-limiting dissections, and the thrombosis in myocardial infarction (TIMI) flow grade is 3, (34) as summarized in Figure 3.

## 3 *In silico* analysis of DCB

Since DCB are typically deployed after LP with semi-compliant/non-compliant balloons or other devices, their primary purpose is to effectively deliver drug. Structural considerations, such as the material properties of the balloon and its interaction with tissue, influence drug delivery. Thus most of the existing computational models of DCB have focused on the drug delivery aspect or the potential influence of material properties on drug delivery. The ability of DCB to effectively deliver drug is influenced by many factors, such as excipient composition, coating crystallinity, type of drug (23) as well as LP, lesion composition and other patient-specific factors (82). This complex interaction between vessel and DCB is not fully understood, particularly with regard to the key parameters of drug release, transport, and retention. The different components that influence drug transport and retention are numerous and interconnected, such as the presence and extent of specific and non-specific binding sites, lesion composition and arterial tissue heterogeneity, blood flow and coating adhesion, to name a few (82). In this section, the literature focusing on drug delivery modelling is reviewed. Studies that have focused on structural considerations are then summarised.

### 3.1 Modelling drug release from DCB

#### 3.1.1 Mechanisms of drug transport and retention

When modelling drug release from DCB, it is crucial to draw upon the extensive literature available on drug release from DES, as it provides valuable insights and foundational knowledge. Drug release from durable polymer coated DES has predominantly been modelled as a diffusion-driven process (83), where drug diffuses through the polymer and into the adjacent arterial tissue. However, several more complex models have been developed to include processes such as dissolution and polymer degradation, depending on the nature of the drug encapsulation and materials (83). It is important to understand that the key difference between drug delivery from a DCB compared with a DES lies in the drug delivery window: DCBs are only in contact with tissue for a very short period of time (around 60 s), whereas DES deliver drug over a period of weeks to months. As a result, DCB tend to have higher drug loading and faster release kinetics. The precise mechanisms of drug transfer and retention from a DCB into arterial tissue are not yet completely understood.

A combination of the balloon properties, drug and excipient are likely to dictate the drug transfer mechanism. For example, it is probable that when in contact with tissue, the balloon delivers drug via a diffusive mechanism, similarly to DES, with diffusion dependent on the drug-excipient coating properties. Drug transport within the arterial wall will also likely be temporarily enhanced due to greater advection as a result of balloon deployment at high pressure, imparting contact pressure on the tissue at a magnitude dependent on the balloon and wall material properties (84). However, there is also evidence to suggest that the coating matrix of the balloon itself may be transferred to the tissue during deployment, subsequently acting as a reservoir for drug to be delivered over a sustained period of time (23). In cases where the drug is loaded on the balloon in a crystalline form, drug dissolution may also contribute to the overall drug release and retention mechanism.

To date, the majority of commercial DCB have been coated with paclitaxel, but limus-coated balloons are becoming increasingly attractive, in part because of a perceived advantageous safety profile, amid concerns regarding very late retention of paclitaxel in arterial tissue. However, limus compounds are less lipophilic than paclitaxel, posing the challenge of controlling delivery and retention of drug such that sufficient concentrations are retained in the arterial wall for a sustained period of time. Enhancement of uptake, retention and sustained availability of dissolved limus drugs, particularly in the context of DCB, are the subject of intense research activity. For example, the SELUTION SLR${}^{\mathrm{TM}}$ DCB utilises MicroReservoir technology to deliver sirolimus (SRL) loaded biodegradable polymer particles to the tissue (85), while the Biolimus A9${}^{\mathrm{TM}}$ utilises Biolimus—a derivative of sirolimus with enhanced lipophilicity (86).

When the drug enters the arterial wall, the drug is known to undergo anisotropic diffusion and advection with specific and non-specific binding processes playing a significant role in the transport process. Experiments of bulk drug uptake into tissue have found that, in equilibrium, paclitaxel and limus compounds distribute differently within the arterial wall. For example, paclitaxel has been shown to deposit preferentially in the intimal and adventitial layer due to the presence of drug-specific binding with intracellular tubulin (23, 82). Sirolimus is more uniformly distributed in the media and adventitia. The capability of hydrophobic drugs such as paclitaxel and sirolimus to remain resident for an extended time period at high concentration in the arterial tissue determines efficacy. Drug retention is governed not only by lipophilic partitioning effects but also by specific and non-specific binding to intracellular and matrix proteins. In particular, the efficacy of sirolimus DES has been associated with prolonged saturation of specific receptors (87).

While most studies (experimental and *in silico*) have considered healthy arteries, it is likely that the presence of atherosclerotic plaque will influence drug uptake and retention (23, 82, 88). In particular, the presence of lipid-rich environments may decrease drug affinity since lipid pools can displace drug-specific binding sites (88). This is important, since lipid-rich arteries and lesions have been associated with increased neointimal growth (89). Fibrous lesions, on the other hand, are rich in elastin and non-specific binding sites that can promote drug uptake, while haemorrhagic lesions can promote drug uptake thanks to increased permeation and transluminal transport. Calcium acts as a significant barrier to drug transport, but may also entrap drug and create a local surplus concentration (82). Understanding drug transport and retention in diseased atherosclerotic tissue is particularly relevant in DCB applications, given the short time window for drug delivery.

#### 3.1.2 *in silico* modelling of DCB drug elution: state of the art

The overwhelming majority of computational models of devices used in PCI focus on DES. There are a handful of models of DCB, considering both peripheral and coronary applications, each with their own set of simplifications and limitations summarized in Table 3. Of these existing articles, eight developed models in a 2D environment (84, 90–96) with only one study considering a 3D (97) arterial model and two developed in a 1D geometry (98, 99). A clear advantage of 1D approaches is the possibility of obtaining either analytical, semi-analytical (99) or easily implementable numerical solutions. However, such models necessarily consider effective or lumped processes/parameters which are not always easily relatable to physical measurements. At the opposite extreme, while significantly more computationally demanding, 3D models have the potential to provide insights into the influence of patient-specific anatomies, although the only 3D model developed to date in the context of DCB has utilised a highly idealised geometry with a simplified calcified plaque. Providing some middle-ground between these two extremes, 2D axisymmetric models have proven popular, but are unable to be applied to patient-specific geometries. The existing models are summarised below, categorised by their dimensional representation. For brevity, full details of the mathematical equations and numerical implementations have been omitted. The reader is referred to each individual study for more details of these aspects. However, those new to the field may find the concise review by McGinty (83) a useful introduction to the fundamental equations governing arterial drug delivery and retention.

**Table 3:** Summary of existing *in silico* models of DCB drug delivery, transport and retention in tissue.

Reference	Dimensions	Peripheral/coronary	No. of arterial layers	Drug source	Advection included	Binding model	Disease included	Drug type
Jain (99)	1D	Coronary	3	Constant	Yes	One phase	No	Sirolimus
Tzafriri (88)	1D	Coronary	1	Flux	No	Two phase	No	Paclitaxel
Kolachalama (90)	2D	Peripheral	1	Flux	No	One phase	No	Zotarolimus
Kolandavelu (91)	2D	Coronary	1	Flux	No	One phase	No	Zotarolimus
Anbalakan (92)	2D	Coronary	1	Flux	Yes	Two phase	Yes	Sirolimus
Anbalakan (93)	2D	Coronary	3	Flux	Yes	Two phase	Yes	Sirolimus
Sariffudin (95)	2D	Coronary	2	Flux	Yes	One phase	Yes	Paclitaxel
Mandal (94)	2D	Coronary	2	Flux	No	One phase	Yes	Paclitaxel
Sariffudin, Mandal (96)	2D	Coronary	2	Flux	Yes	Two phase	Yes	Sirolimus
Escuer (84)	2D axisymmetric	Coronary	3	Flux	Yes	Two phase	No	Sirolimus & Paclitaxel
Colombo (97)	3D	Peripheral	1	Constant	No	One phase	Yes	Paclitaxel

#### 3.1.3 One-dimensional models

**Figure 4:**
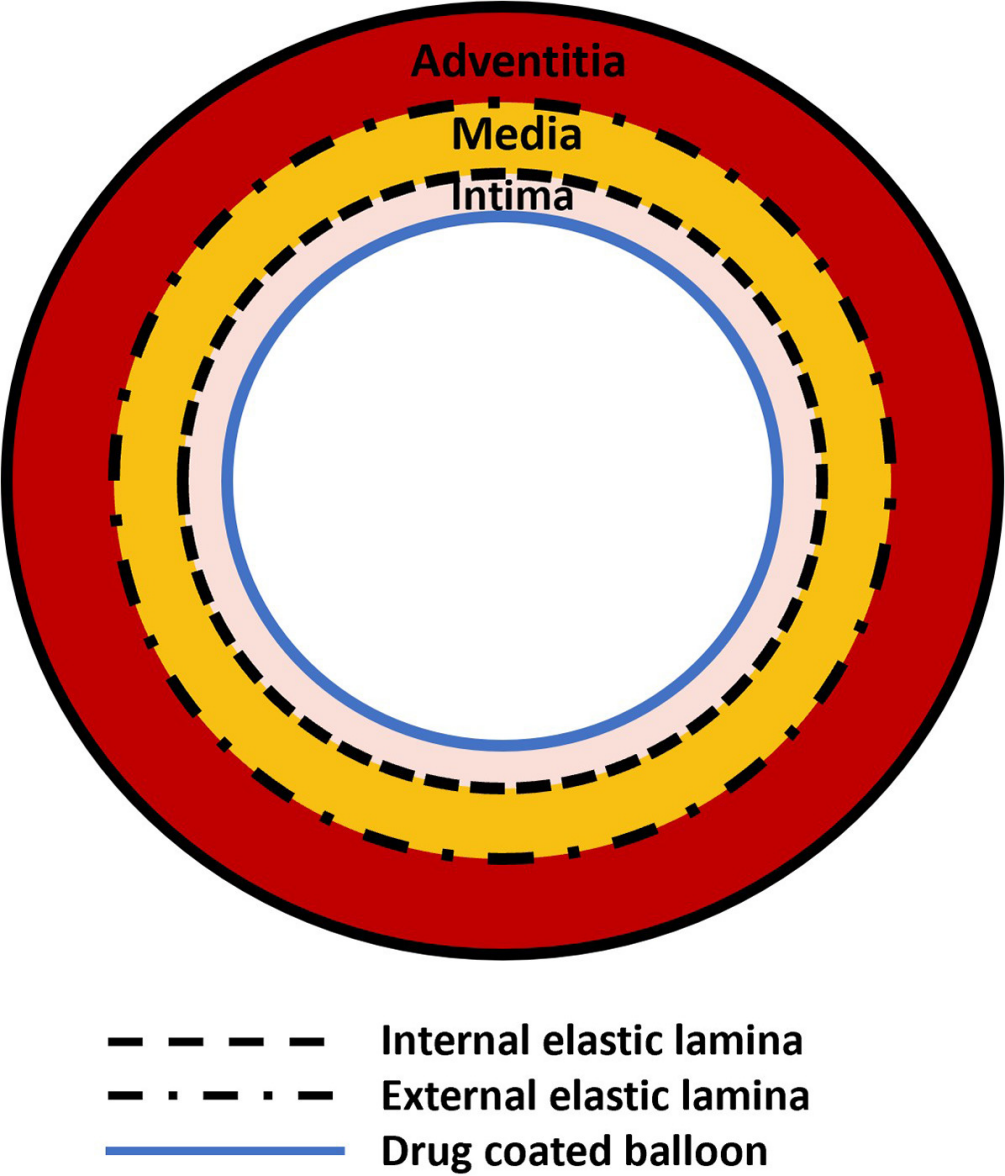
Schematic of the model based on the work by Jain et al. (99). A balloon surface is in contact with a three-layer artery.

Jain et al. (99) proposed a one-dimensional axially-symmetric model (Figure 4) to describe drug release from a DCB and subsequent transport within a multi-layer cylindrical artery. A linear advection-diffusion-reaction equation was proposed for an arbitrary number of layers. Two distinct stages were considered: the first corresponding to the period during which the balloon was in contact with the tissue and the second corresponding to the period post-balloon contact. A closed-form analytical solution was developed with results presented for a three layer artery including the intima, media and adventitia. Other than the geometrical idealisation, a key limitation was the consideration of a linear irreversible binding reaction, a necessary simplification to enable an analytical solution (Figure 4). A sensitivity analysis was performed around the inner and outer boundary conditions, the reaction coefficient and the diffusion coefficients. It was found that the amount of drug delivered during the short application period was influenced strongly by the diffusion properties of the innermost layer, while on a longer timescale the inner and outer boundary conditions, expressed in terms of non-dimensional Sherwood numbers, played a key role in determining the drug content within the artery. It was concluded that, under typical conditions, a relatively low amount of the DCB drug mass is delivered to the tissue.

Tzafriri et al. (98) proposed a one-dimensional model to study how dissolution influences paclitaxel retention in tissue following delivery from a DCB. Drug transport followed a reaction-diffusion model, but differently from Jain et al. (99), advection within tissue was neglected and binding was described as a reversible nonlinear process with two distinct phases (drug bound in the extracellular compartment and within cells to specific binding sites). Moreover, internalisation of drug within cells was modelled prior to specific binding. The source of drug was modelled via a flux boundary condition that described dissolution of drug adhered to the lumen following balloon deployment. The authors demonstrated that the rate of paclitaxel coating dissolution was limited by its mass transfer coefficient, enabling a pseudo-steady state approximation of the diffusive flux and the concentration of soluble drug at the coating-tissue interface. The key finding of the work was that the dissolution mechanism provided a means of sustained drug retention and protected against diffusive clearance of the drug.

#### 3.1.4 Two-dimensional models

**Figure 5:**
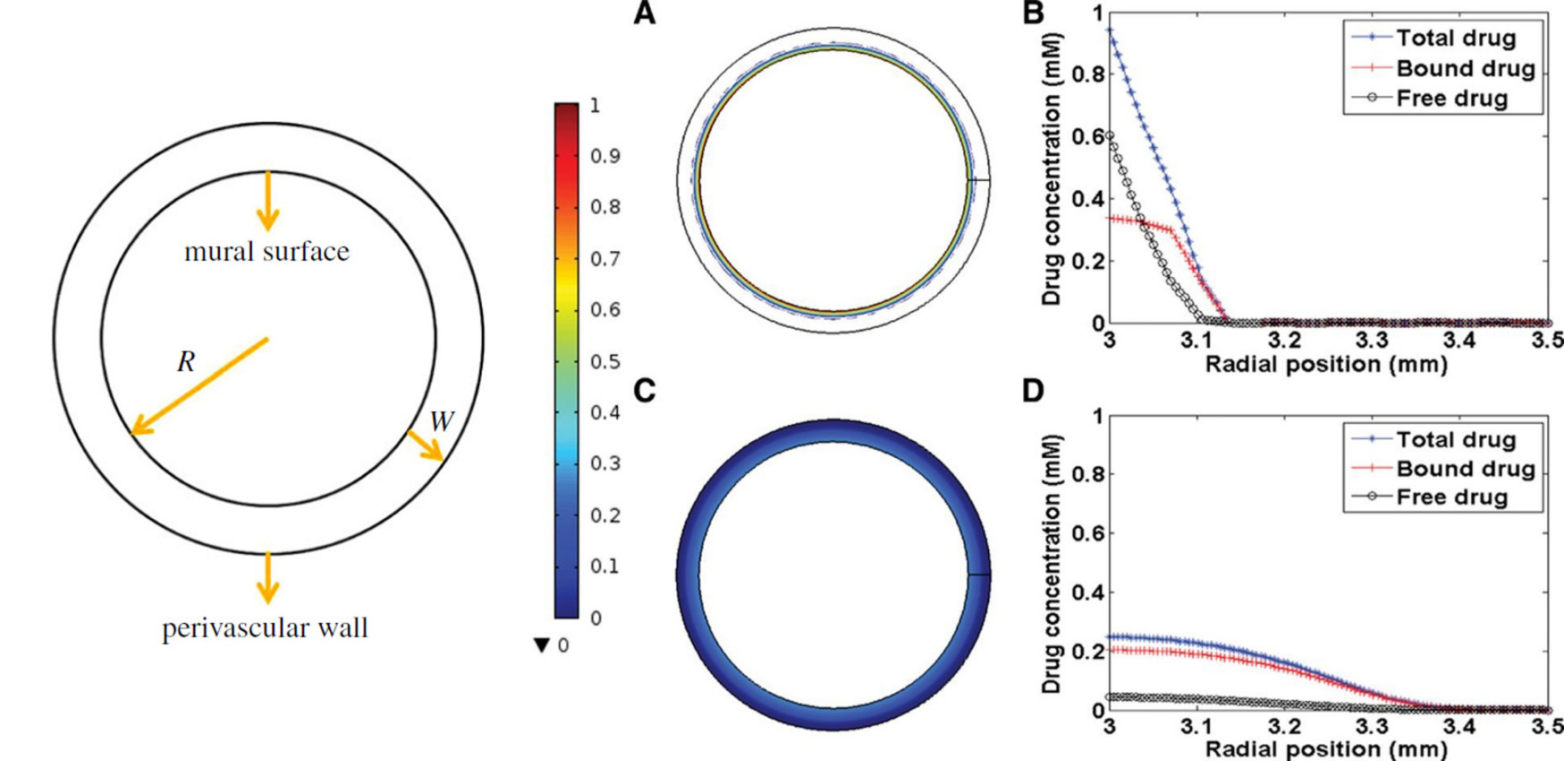
Schematic representation of cylindrical model (left) (91) and drug distribution: **(A**, **B)** total drug concentration at 30 s, **(C**, **D)** total drug concentration at 5 min (90), obtained under the terms of the Creative Commons Attribution License http://creativecommons.org/licenses/by/4.0/.

Kolachalama et al. (90) developed a study to quantify drug distribution after DCB deployment in peripheral disease using *in vivo* and *in vitro* experiments combined with computational modelling. The computational model utilized a one-layer, 2D hollow cylinder cross-section with homogeneous tissue characteristics, in a configuration similar to *in vivo* bench tests (Figure 5). Zotarolimus transport in the tissue was modelled via a single isotropic diffusion coefficient, estimated via the experiments, and a non-specific reversible binding mechanism. A drug influx based on exponential decay was prescribed, parameterized based upon experimental data, while two extreme boundary conditions were considered to model the effect of blood flow in the lumen: in one case the mural-adhered drug was considered insensitive to the blood flow (zero-flux condition), while in the second case the adhered drug was considered to be completely washed out by blood (sink condition). Two theories were formulated on the mechanism of drug transfer and sustained retention: (i) transport in the arterial wall is governed by diffusion after balloon deployment, while distribution and retention are governed by binding of soluble drug to the arterial tissue and (ii) a large portion of drug and excipients distribute in the artery as microparticles and it is their solubilization that determines tissue retention.

A similar geometrical model was used in Kolandaivelu et al. (91) (Figure 5). Here, two computational models of PCI intervention were studied: one simulating drug release from a DCB in a 2D, time-dependent model, and the second simulating drug release from a DES in a 3D model. Again, a diffusion-reaction equation was used to describe drug transport from the DCB, with constant diffusivity and reversible binding to tissue sites, with drug release from the DCB modelled via a flux condition during balloon expansion. The article was, however, focused on using supervised machine learning to reduce simulation time, hence a simplified environment was used and drug distribution was studied in relation to mesh coarseness.

The 2D cylindrical cross-section geometry was also adopted in later work by Anbalakan et al. (92) and developed to evaluate the influence of atherosclerotic lesions on sirolimus drug uptake during DCB therapy. Two vessel configurations were investigated, one representing a healthy vessel and one representing a diseased arterial wall with plaque inclusion. Drug transport was described using an advection-diffusion-reaction equation where the initial DCB drug concentration value was adjusted to account for experimentally observed tracking loss. Finally, the input drug was modelled as a flux based on experimental data that, after balloon removal, was dropped to zero in the lumen. This simulated the effect of blood flow that efficiently washes away any residual. Differently from Kolandaivelu et al. (91) and Kolachalama et al. (90), two non-linear phases of binding were used to model drug retention in the tissue. The plaque was modelled through a parametric characterisation based on porosity to evaluate transport in different material models. In a later study by Anbalankan et al., a new 2D arterial model was developed that took into consideration the multi-layer nature of the arterial wall (93). In this case the intimal layer was developed to represent either the healthy vessel tissue or the lesion (atheroma). The properties of the lesion were varied to represent soft to hard plaques. Each material was modelled with a different porosity to characterize different tissue composition.

In their earlier work (92), it was observed that porosity affects both bound drug saturation limit and duration of saturation. In the presence of a lower porosity (corresponding to calcified plaque), even when balloon contact is prolonged, drug uptake was not comparable to that of healthy arterial tissue. In their later study (93), the opposite trend was observed in the case of tissues characterized by higher porosity, where the whole intimal layer porosity was subjected to changes. When high porosity values were modelled, a higher concentration of drug was observed in the intimal layer (42%) when compared to the healthy configuration (38%), showing that, based on material plaque properties, the drug transport can be enhanced due to either higher diffusivity coefficients or higher density of specific binding sites in tissue. These results suggest that modelling the disease components strongly impacts on drug elution during DCB application, and subsequent transport and distribution within the lesion. Morphology, composition, and degree of calcification play important roles in drug transport and, ultimately, DCB therapy effectiveness. It was observed that drug concentration accumulated in the intimal layer (93), moving through different layers in time. After 30 min, 44% of sirolimus was found in media and 23% in the adventitia when a hard plaque model was used. In the case of a soft plaque model, 42% of sirolimus accumulated in the lesion (located in the intimal layer), higher than the percentage of drug in healthy tissue 38%. The authors indicated that these results, combined with the fast saturation of specific and non-specific binding sites, show that increasing drug dose on the DCB does not significantly increase drug uptake or retention by the arterial tissue. A general decrease of 15.5% in sirolimus uptake was observed at 30 s inflation time when compared to higher application times. Finally, Anbalakan et al. also observed that when a low porosity material model was utilized, even when exposed to a prolonged application time, drug uptake could not compare to that in healthy tissue and remained lower in concentration. In their study, it was suggested that, rather than increasing inflation times to increase drug uptake, it may be possible to identify an optimal therapeutic window for DCB expansion that would lead to better procedure outcomes. The idea is that this therapeutic window would be strictly related to coating and release profile of the matrix and that a better drug distribution could be achieved without simply increasing balloon application time.

In all the aforementioned studies, no patient-specific approach was considered when modelling the vessel geometry. Mandal et al. (95) obtained a 2D longitudinal arterial geometry from a single virtual histology intravascular ultrasound (VH-IVUS) patient-specific image. A 2-layer model was developed: the first layer accounted for the intimal region segregated by plaque composition: fibrous cap, fibrofatty lesions, necrotic core, calcified lesions, and healthy tissue. The second layer comprised the medial and perivascular region. Paclitaxel transport was modelled as a diffusion driven process, while drug binding to tissue was modelled as a single-phase reversible process. This model was developed further in a subsequent study by Sariffudin et al. (94) where attention moved towards the effects of interstitial flow (plasma convection and bound drug internalization) on transport and retention of drug from the DCB. The process of internalization of drug and lysosomal degradation during endocytosis was mathematically modelled. In both studies, a mean value for arterial thickness was evaluated and drug delivery was modelled imposing a flux boundary condition. The influence of luminal boundary conditions was observed to have a great impact on drug retention in the arterial layers. In Mandal et al. (95) three luminal boundary conditions were tested at 25 h: a zero-flux condition meaning that there was mural-adhered drug, a hybrid wash-out condition and a zero-concentration condition meaning that blood was very efficient at wash-out. In this work, the residual free drug concentration in the tissue was 1.9% in the case of the zero flux condition compared to 0.21% in case of blood wash out, while the bound drug concentration was found to be 41% and 10% respectively after the deliver by 25 h. Sariffudin et al. (94) observed that convection promoted rapid saturation of binding sites in a manner that depended on the boundary conditions imposed at the lumen, with binding site saturation ranging from 15.4% to 98.3% at 25 h for the two extreme boundary conditions of zero concentration and zero flux respectively. They also observed that the lumen boundary conditions influenced the internalization of drug showing concentrations values of internalized drug: 58.4% and 100% of the total drug delivered at 25 h for zero concentration and zero flux boundary conditions, respectively.

Subsequent work from Sariffudin and Mandal (96) used a 2D radial cross-section of a patient-specific coronary plaque based again on VH-IVUS images. Three different cross-section models where used: the first taking into consideration patient-specific variations of the plaque, comprising of five plaque elements (healthy, fibrous, fibrofatty, necrotic core and dense calcium); the second model named as “hard model” was built only using healthy, necrotic and calcified tissue; finally, the “soft Model” was composed of healthy, fibrofatty and fibrous material models. Transport was modelled using an interstitial plasma flow through porous tissue assigned with a fixed velocity, while drug binding was modelled as two-phase and reversible. Once again, two extremes where adopted as boundary conditions in the form of no-flux and sink conditions. In this study, it was observed that various tissue compositions in arterial and plaque microstructure influence and modulate endovascular delivery. Sirolimus may have a higher diffusivity in regions such as fibrofatty and fibrous and lower diffusivity in calcified and necrotic tissue, hence the “soft” model displayed a maximum drug uptake of free sirolimus and more evenly distributed free drug in the tissue. Receptors and matrix bound drug concentrations were highest in the soft plaque when compared to other tissue models, with a receptor occupancy of 50% in 1 h, which was around 22% higher than in the healthy reference model (receptor occupancy in the hard plaque was only 7%). Another aspect taken into consideration was plaque components and their positioning. Four new models were implemented to study different aggregations of necrotic and calcified plaque that typically display little or absent drug uptake. It was observed that placement closer to the lumen interface and/or clustering has a substantial impact on drug delivery and its retention.

#### 3.1.5 Two-dimensional axisymmetric models

**Figure 6:**
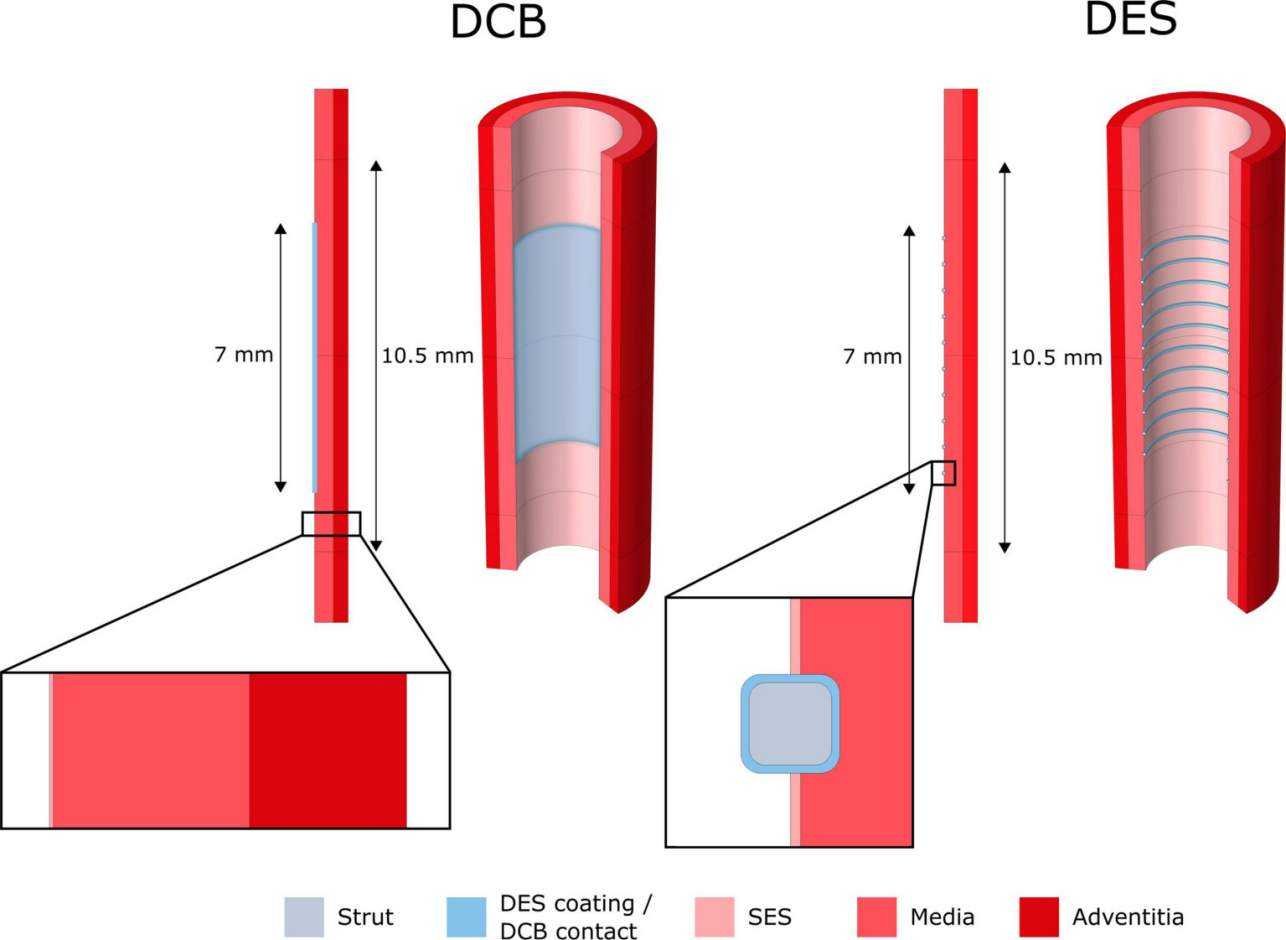
2D-axisymmetric model of drug elution from DCB and DES into a cylindrical, three layered arterial wall. SES, sub-endothelial space. Obtained from (84) under the terms of the Creative Commons CC-BY license.

A 2D-axisymmetric geometry in a multilayer arterial wall was employed by Escuer et al. (84) to simulate drug delivery from stents and different types of balloon platforms. Various pressure conditions were studied during DCB application and comparisons were made between the delivery of sirolimus and paclitaxel. Two nonlinear phases of binding, together with advection-diffusion, were used to describe drug transport and absorption through the vessel wall. A three-layer arterial wall was employed including adventitia, media and sub-endothelial space (Figure 6) and each layer was assumed to have different transport properties. To model drug delivery from the DCB, similarly to the previously described 2D models, a flux condition was assumed on the lumen wall, and after delivery an infinite sink condition was applied on the lumen surface to simulate wash-out.

Two interesting parameters were explored in this work: inflation pressure and balloon application time. Escuer et al. demonstrated that pressure plays an important role in drug delivery from the DCB as it is the pressure gradient that drives the advective term of drug transport. In the presence of high-pressure values during balloon deployment, the advective term was seen to significantly increase, therefore delivering the drug deeper into the arterial wall. Consequently, a lower quantity of drug was exposed to luminal washout leading to an increase in drug content (DC) in the tissue and a slower decline of DC in time. It was further noted that higher DCB application times corresponded to higher sustained saturation of specific binding sites (% SBSS) for the drug sirolimus, important since there is general consensus in the literature, at least for sirolimus, that saturation of specific binding sites dictates efficacy of endovascular devices (87). Escuer et al. showed that more receptors were saturated for longer when a higher dose of sirolimus was modelled, in agreement with Sariffudin et al. However, it was also demonstrated that once the binding sites are saturated, the excess drug quantity will have no effect and will be either washed away or degraded, in line with the findings of Anbalakan et al.

**Figure 7:**
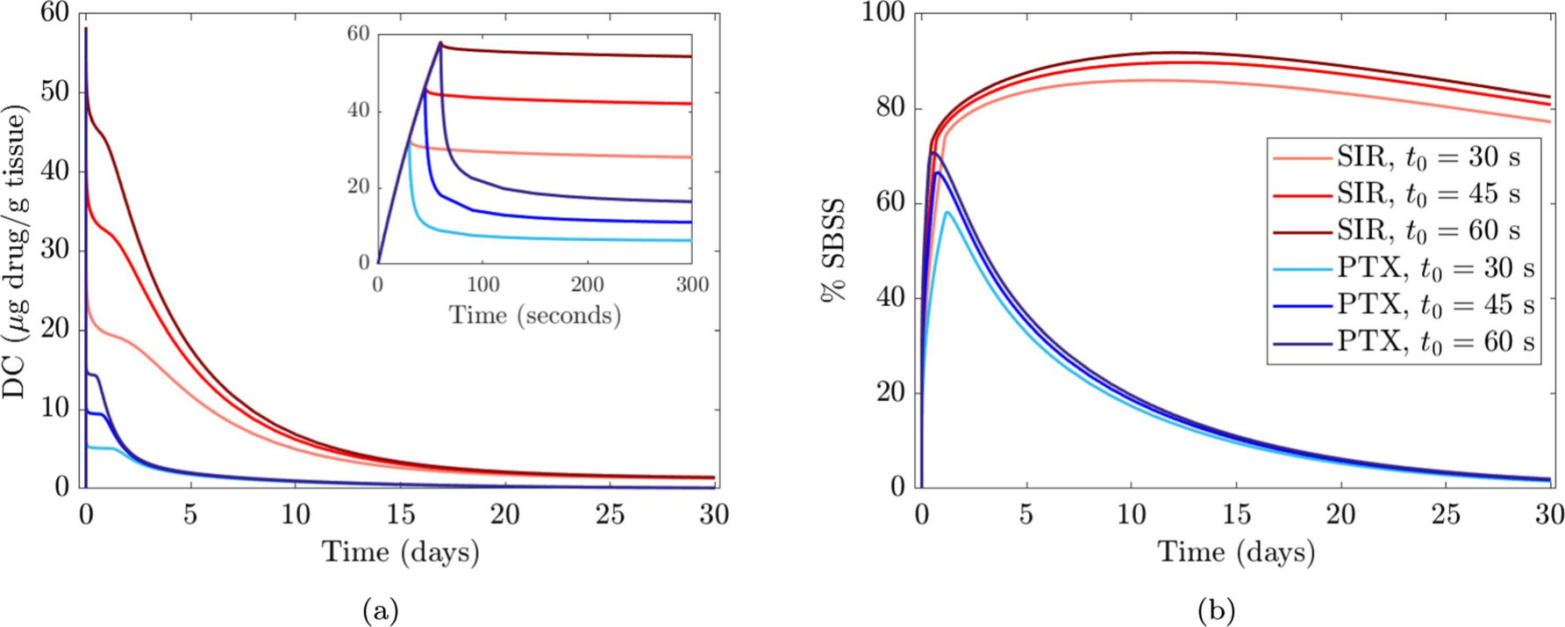
Comparison between sirolimus (SIR) DCB and paclitaxel (PTX) DCB at different balloon application time values (30, 45, and 60 s). **(a)** Drug content (DC) vs. time. **(b)** % specific binding site saturation (%SBSS) vs. time. Obtained from (84) under the terms of the Creative Commons CC-BY license.

Until recently, the majority of commercial DCB were coated with paclitaxel, thus many of the studies considered modelled paclitaxel as the reference drug, with sirolimus and zotarolimus the other choices modelled. Only Escuer et al. presented a comparison of drug uptake and transport dynamics between paclitaxel and sirolimus, utilising the limited drug binding parameter values available in the literature. When the initial drug dose and delivery mechanisms were assumed identical for paclitaxel and sirolimus, the DC peaks were equal in the early time frame post DCB deployment. However, differences in transport and binding kinetics of the drugs in the arterial tissue led to differences in the decline of the DC curve, with a faster DC decline in the case of paclitaxel compared to sirolimus, (Figure 7a). Paclitaxel was observed to have lower peak %SBSS with a faster decline from the peak, therefore, with the same delivery sirolimus was retained for longer in the tissue (Figure 7b). While more related to drug retention than efficacy, non-specific binding site saturation (%NSBSS) peaked at only 30% and 25% for sirolimus and paclitaxel, respectfully. Finally, given their comparison of two DCB with vastly different drug doses and release kinetics, Escuer et al. noted that there seemed to be ample room for optimisation of the drug dose and release kinetics to achieve maximum efficacy while reducing drug wastage.

#### 3.1.6 Three-dimensional models

**Figure 8:**
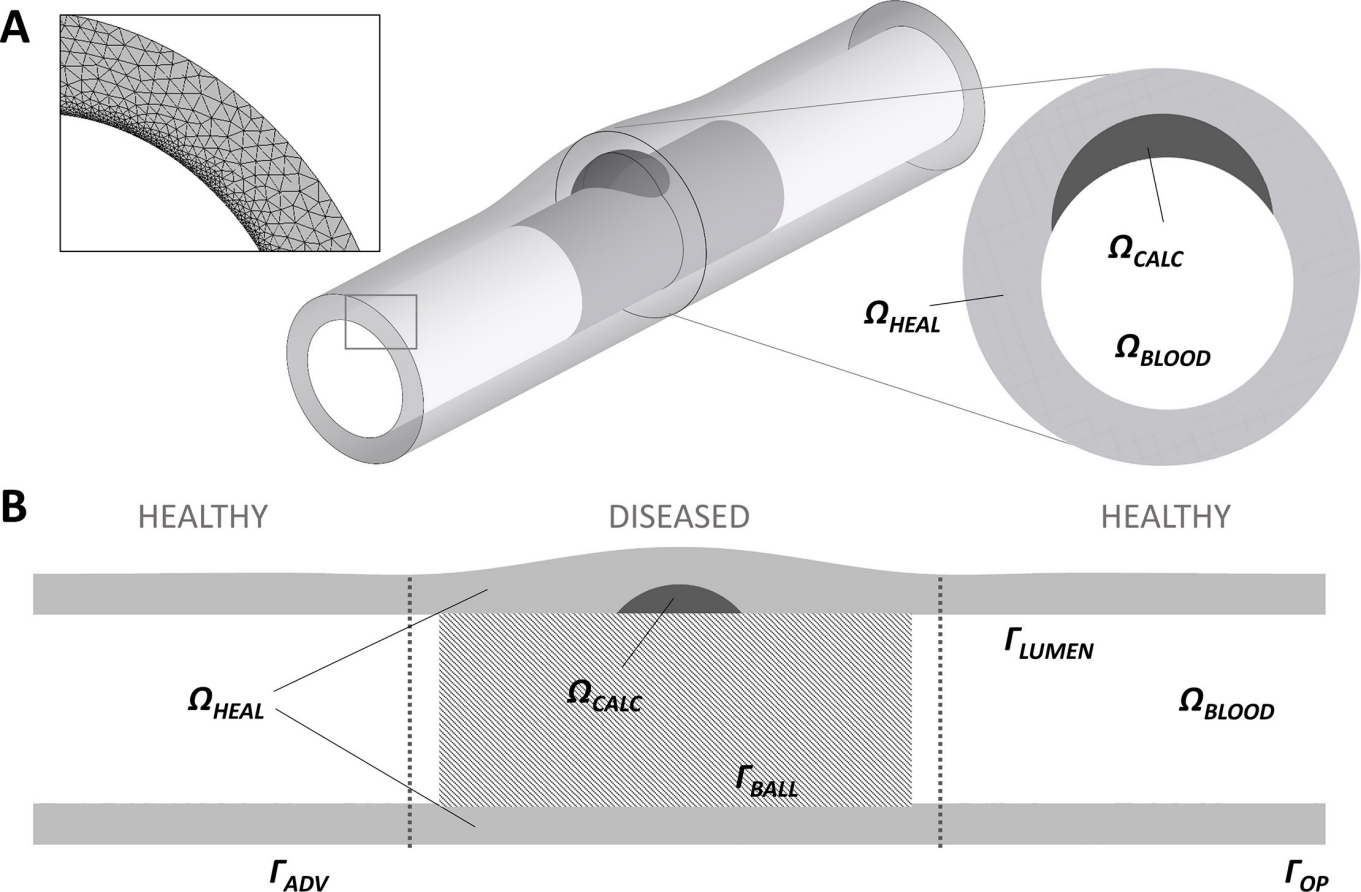
A schematic of the 3D arterial model developed by (97), obtained under the terms of the Creative Commons Attribution License, including healthy tissue and a calcification **(A)** with a close up on circumferential meshing and plaque model. **(B)** Longitudinal cross-section of the model highlighting the computational domains: healthy domain $\Omega_{\mathrm{HEAL}}$, calcific plaque domain $\Omega_{\mathrm{CAL}}$, lumen domain $\Omega_{\mathrm{BLOOD}}$, adventitia wall $\Gamma_{\mathrm{ADV}}$, wall in contact with the balloon $\Gamma_{\mathrm{BALL}}$, lumen wall $\Gamma_{\mathrm{LUMEN}}$, vessel wall lateral opening $\Gamma_{\mathrm{OP}}$.

To date, the only 3D model of DCB drug release and tissue retention has been provided by Colombo et al. (97). A 3D model of a diseased superficial femoral artery was designed to have homogeneous tissue characteristics except for the presence of a calcification (Figure 8). Drug delivery from the DCB was approximated as a constant source of drug during the period of application. A transient linear reaction-diffusion equation was utilized to model drug transport through the vessel wall, with reaction described via a single phase, non-linear, reversible saturable binding term, while advection was neglected. After balloon deflation and removal, the effect of blood flow on drug was modelled as a zero concentration boundary condition (BC) on the lumen of the vessel. Differently from all the aforementioned models, here the possibility of drug coating adhering to the mural wall was also considered (in a simplified way) by prescribing an exponential decay of drug at the lumen boundary following DCB removal.

**Figure 9:**
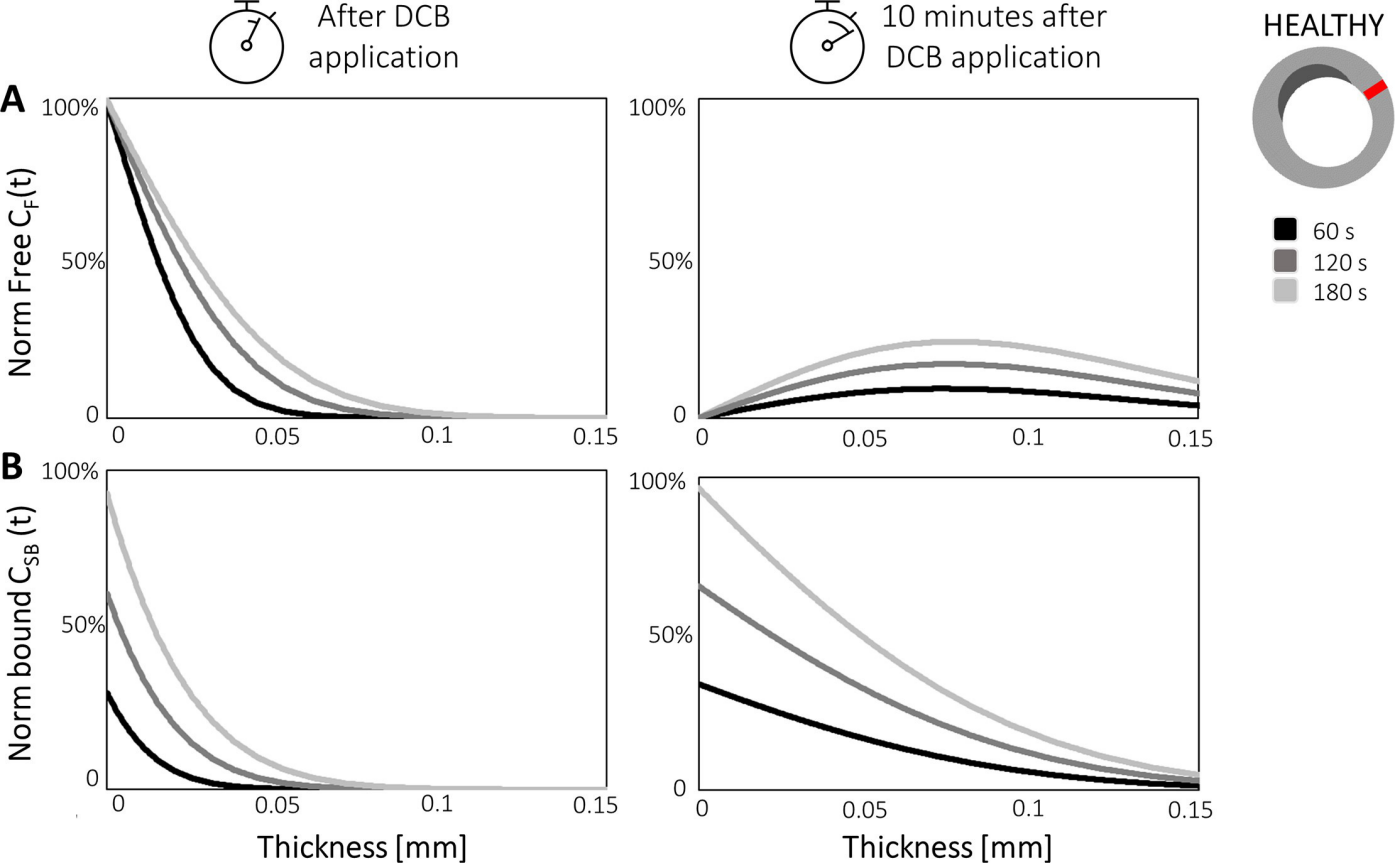
Free drug *C_F_* **(A)** and bound drug *C*_SB_ **(B)** concentrations are plotted over artery thickness in the healthy portion of the vessel wall (red) after different balloon application times (60, 120, and 180 s) in Colombo et al.'s model. The two concentrations are plotted during DCB application (left graphs) and 10 min after the balloon has been deflated (right graphs). Obtained from (97), under the terms of the Creative Commons Attribution License.

Colombo et al. observed that modelling coating retention via the exponential decay term resulted in free drug concentrations around 3.5-fold higher and bound drug concentrations around 4-fold higher than in the case of drug wash-out from the lumen. Since they were considering application to a peripheral artery, Colombo et al. simulated a longer inflation time (180 s) and found that this resulted in 30% higher free drug concentration and 3-fold higher bound drug concentration at the end of the DCB application, compared with the 60 s application. This finding is in agreement with several of the previous works, where increasing the DCB application time was found to strongly influence drug uptake. Moreover, the concentration of free drug at 180 s was four-fold higher in healthy tissue compared to the case of the diseased tissue (Figure 9), as a result of the impenetrability of the calcium. Another interesting aspect of Colombo et al.’s study was the consideration of sequential balloon applications. Here, single balloon inflations and double consecutive balloon inflations (60 s with a 5 min interval between inflations) were tested with an equal total exposure time of 120 s. It was observed that free and bound drug concentrations were similar in healthy arterial tissue between single and double balloon applications, however a lower free drug concentration was observed in the case of the double balloon application. The concentration of bound drug was also found to be lower (by 38%) in the proximity of the lumen wall following the double application. These results are probably explained by the 5 min time interval between the inflations facilitating drug wash-out.

### 3.2 Structural considerations

#### 3.2.1 Impact of structural mechanics of the excipient and balloon on DCB drug transfer

Each of the models of DCB drug elution and tissue retention presented in Section 3.1 assumed that drug was transferred to the artery via a temporary boundary condition (either prescribed flux or concentration) upon contact with the tissue. However, they neglected any mechanical interaction (e.g., deployment and contact) between the DCB and the tissue that may influence drug transfer and retention.

**Figure 10:**
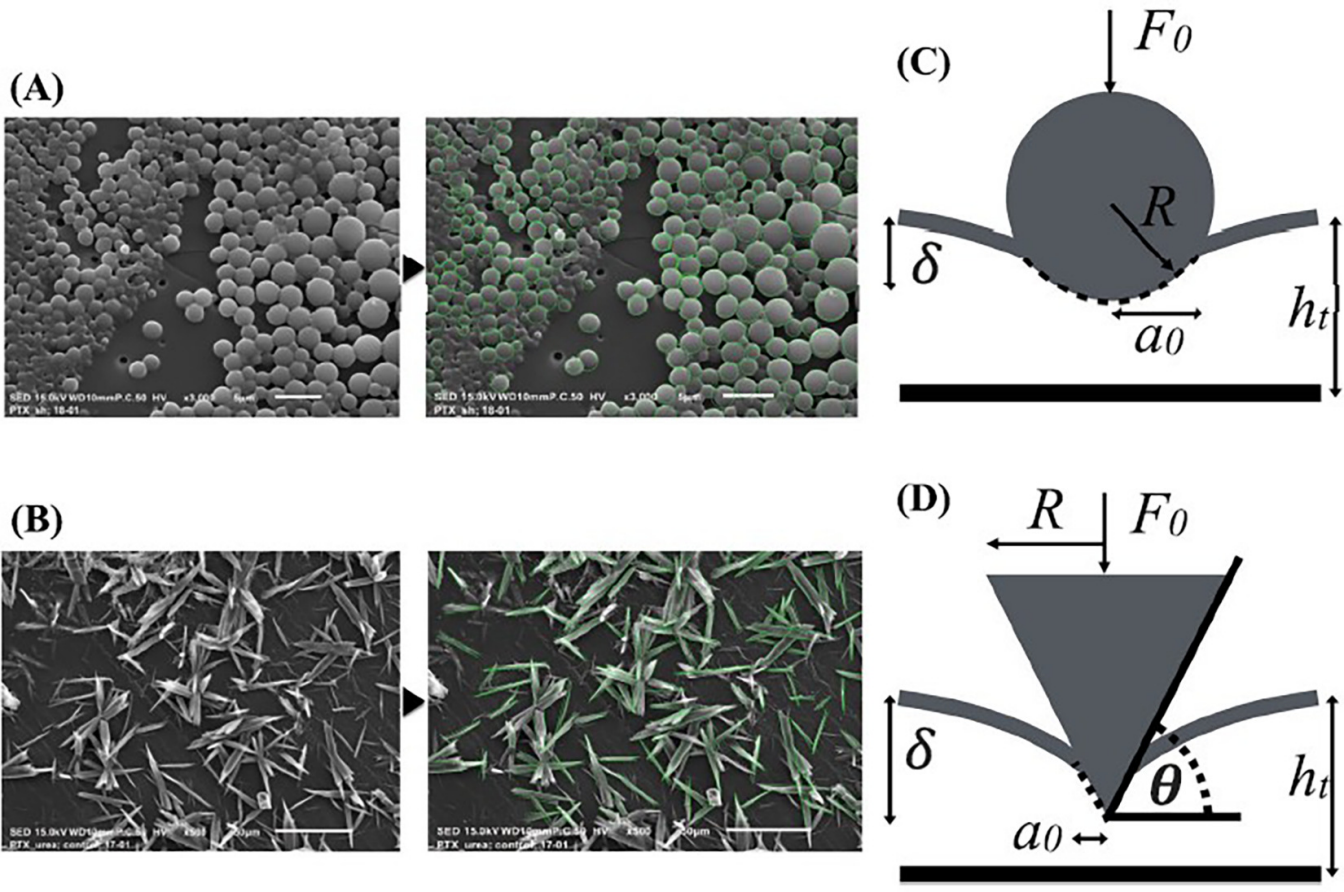
Chang et al.’s SEM images of shellac **(A)** and urea **(B)** with associated contact mechanics models based on Hertz theory: spherical model for shellac **(C)** and conical element for urea **(D)**. Contact radius $a_{0}$, indentation depth δ, radius of the intrinsic spherical element R, resulting force applied by the elements on the arterial wall $F_{0}$, angle between the conical shape and the indented surface θ. Obtained from (100), under a Creative Commons Attribution 4.0 International License.

**Figure 11:**
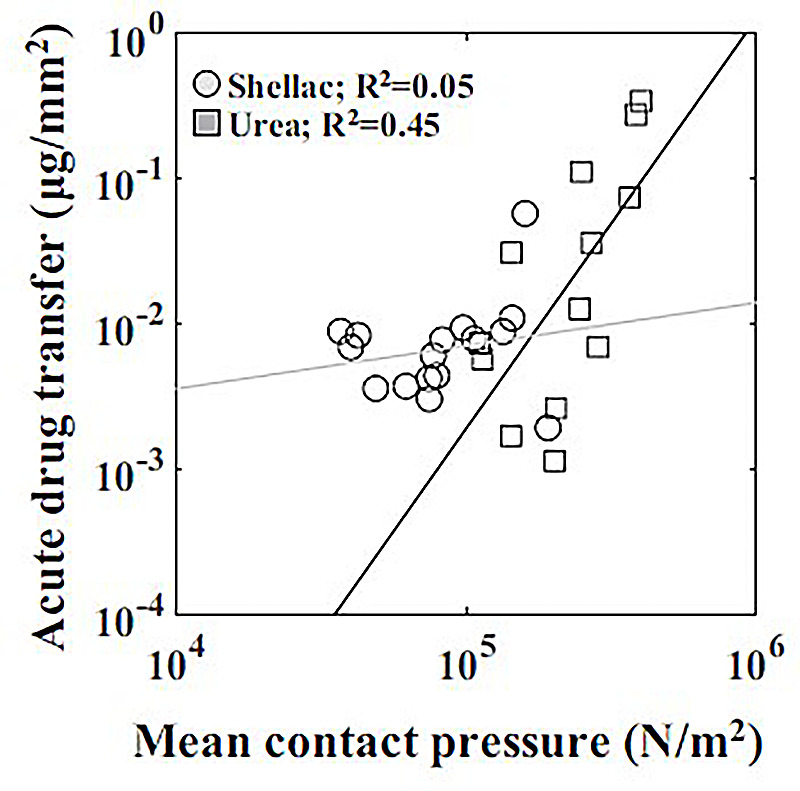
Correlation between acute drug transfer following mechanical testing with mean contact pressure depending on the coating (shellac and urea): a stronger correlation was observed with urea than shellac. Obtained from (100), under a Creative Commons Attribution 4.0 International License.

In study by Chang et al. (100), a series of bench-top experiments and mathematical models were developed to estimate the coating-specific mechanical behaviour between balloon and arterial wall. In this study, two types of coating matrix were analysed on different scales: shellac and urea, mixed with paclitaxel. The microstructure of the two matrix components was obtained through scanning electron microscopy (SEM), and two distinct shapes were identified. Shellac was characterized by a spherical microstructure, while urea appeared to have a needle-like structure that was idealized as a conical shape (see Figure 10). The authors used Hertz theory to model the interaction between coating matrix and arterial wall as an elastic contact problem. A surface contact region was defined for both shapes in terms of the contact radius (see Figure 10). Contact forces were then computed and used to define a mean contact pressure in relation to the mean contact area. It was observed that the mean contact pressure values, as well as the size of the contact region, were statistically different between shellac and urea coatings. Moreover, acute contact drug transfer was measured using liquid chromatography and correlated to mean contact pressure, showing a stronger drug uptake in the urea case (spherical) compared to the shellac coating (conical) (Figure 11), meaning that the unique microstructure of the balloon coating directly influences drug transfer to the artery.

Subsequently, Tzafriri et al. (101) studied the role of matrix coating properties and balloon forces interacting with the arterial wall. A computational model was developed to study the mechanical properties of coating micro-morphology on the endothelium and a separate model was developed to describe and predict local arterial micro-indentation pressure derived from the coating particles. A SEM analysis was used to correlate coating embedment in the tissue and its distribution after balloon deployment. Three different DCB and their respective coatings were selected and grouped based on their coating morphology: microneedle or amorphous/flaky structure. The model was used to predict the contact stress at the balloon-artery interface; hence a microscopic radial pressure was computed along the contact axis of the balloon. As the balloon expands, the coating is pushed against the arterial wall, causing a micro indentation pressure different from the expansion pressure from the balloon. Therefore, the expansion pressure was considered as an invariant boundary condition at the level of the coating particles, hence driving their micro indentation in the tissue. As in the previous work (100), the structure of the coating particles was modelled with simpler geometries: a micro-cylinder for the microneedles and a flat disk for the flaky/amorphous coating. Again, the pressure for both the microneedle and disk coating were computed together with paclitaxel distribution after balloon deployment. It was observed that the disk particles were able to transmit 80% of the angioplasty pressure whilst the microneedles were found to always amplify angioplasty pressure. Also, the paclitaxel distribution varied greatly depending on the micro indentation pressure and on the spatial component. Together these results demonstrated a connection between the DCB/arterial wall contact forces and the intrinsic shape of the coating matrix microstructure. Different excipients will have different micro surface structure that will ultimately influence drug uptake and pharmacokinetics. Moreover, the coating properties determine a different interaction with the vessel which directly influences the capability of the coating to adhere to the vessel wall and potentially act as a local delivery of drug to the mural surface. Also, balloon inflation is often subjected to inhomogeneities, with the lateral portion possibly subjected to contact with the arterial wall at different degrees than other more central portions. A microstructure that can amplify pressure will be less sensitive to these variances in contact and contact pressure within the balloon, while amorphous/flaky coating will be more sensitive to these local variations of pressure. Local drug transfer will be even more greatly affected in the presence of a diseased vessel, where plaque morphology and composition will attenuate or increase this phenomenon. From the paclitaxel spatial distribution images, it was also possible to observe that drug distribution following DCB deployment was not as uniform as originally believed, with the average tissue coverage of paclitaxel being <18%.

#### 3.2.2 Impact of balloon unfolding and deployment on DCB drug pattern

**Figure 12:**
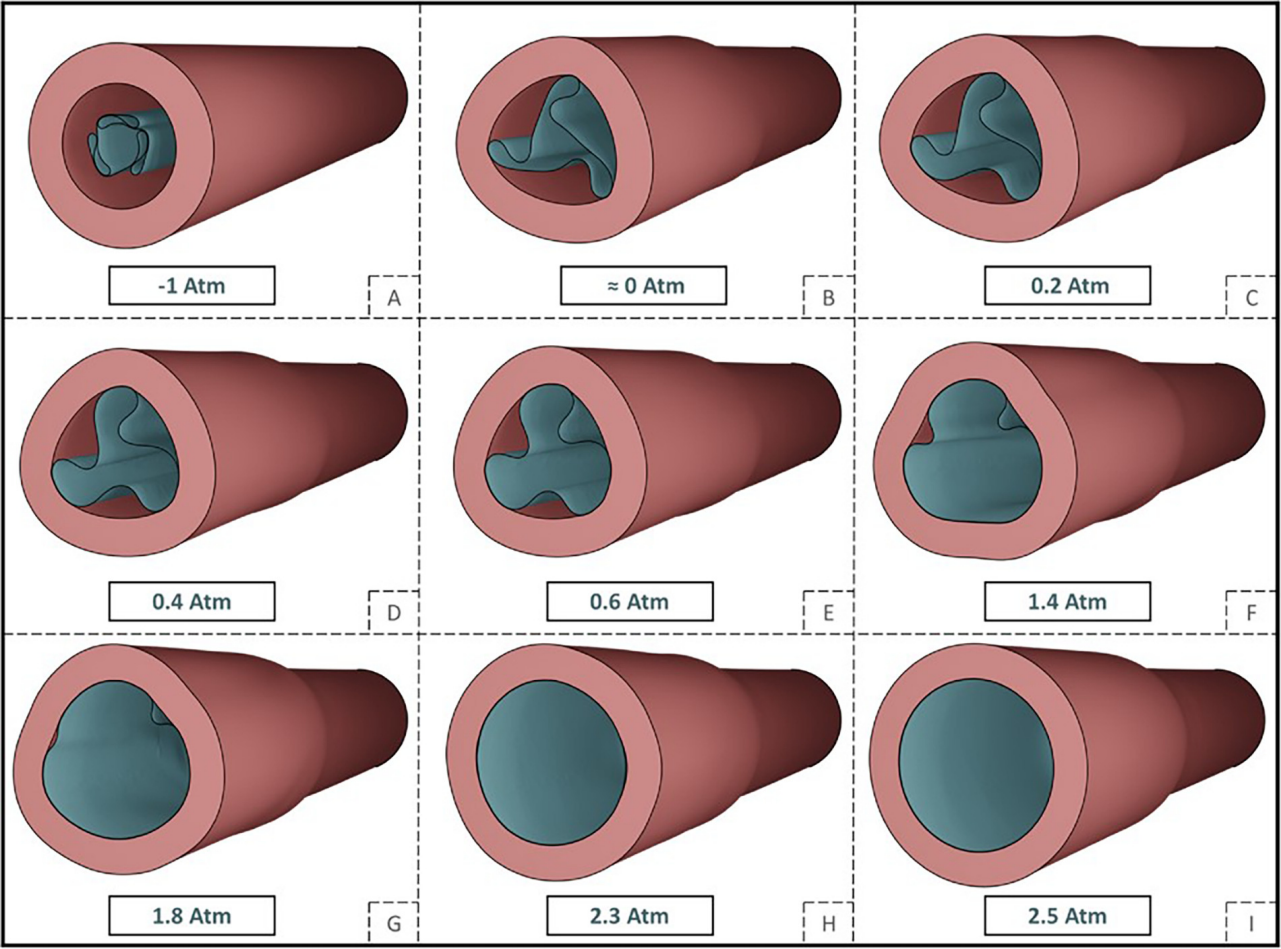
Depiction of a tri-folded balloon unfolding **(A–G)** and deployment **(G–I)** in cylindrical arterial model and relative internal pressure values. Obtained from (102) under a Creative Commons Attribution 4.0 International License.

**Figure 13:**
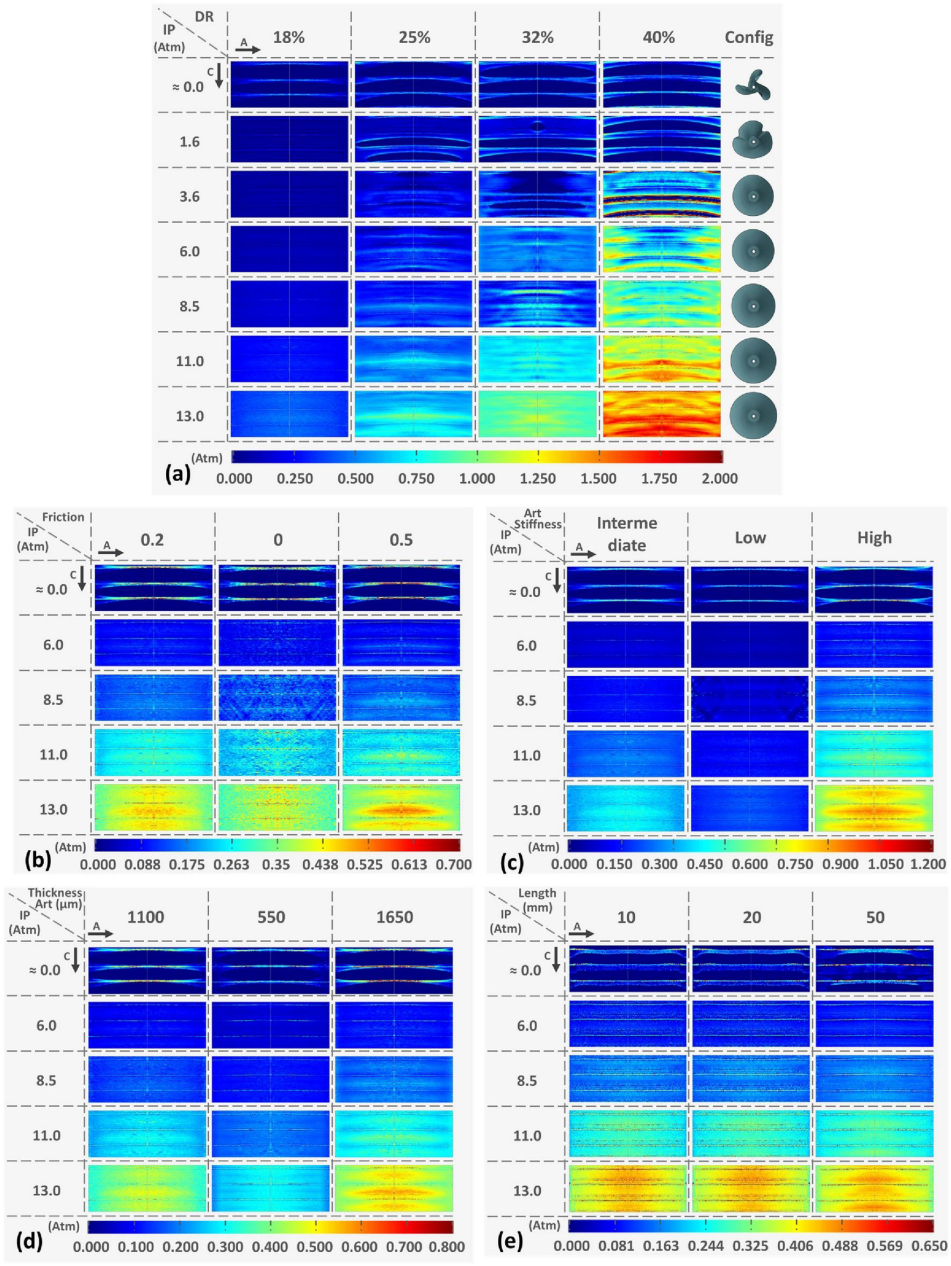
Contact pressure (CP) pattern colour map at increasing internal pressure values and varying parameters: **(a)** Diameter ratio (DR) at 18%, 25%, 32%, and 40% configuration: **(b)** friction penalties of 0, 2, 0, and 0.5: **(c)** increasing vessel stiffness from low to high: **(d)** varying arterial thickness and **(e)** for increasing vessel longitudinal length. Obtained from (102) under a Creative Commons Attribution 4.0 International License.

In a study from Stratakos et al. (102), finite element modelling was used to investigate angioplasty balloon interaction with the arterial wall for peripheral artery disease applications. Based on observations from Tzafriri et al. (101) and Chang et al. (100), that biophysical forces affect coating transfer to the arterial wall, Stratakos et al. focused on understanding the impact of contact pressure (CP) on coating micro-patterns transferred to the wall. A numerical model of a peripheral balloon was created incorporating the unfolding process and contact with the arterial wall (Figure 12). The vessel was modelled as an idealised hollow cylinder with hyperelastic material properties and dimensions based on healthy femoral arteries, while the balloon was modelled as linear elastic semi-compliant using shell elements with variable longitudinal length and thickness. The balloon-vessel interaction was then studied under different procedural conditions accounting for various balloon or vessel characteristics, such as vessel wall stiffness and thickness, balloon length and longitudinal thickness variation, and balloon-to-vessel ratio. For each condition and interaction, CP maps where then generated and validated against data from pig arteries (101). From the computational simulations it was firstly observed that the balloon comes in contact with the vessel wall during the unfolding process. This seemed to determine a non-uniformity in CP patterns that corresponded to the points of contact between the vessel wall and the apex of the balloon folds in the early stages of unfolding. It was then noted that the CP in the vessel seemed to be dependent on the initial contact of the folds during the expansion. This phenomenon was attributed to the influence of the friction coefficient as the balloon has a tendency to rotate. The friction coefficient can be impacted by factors such as coating matrix and structure, vessel properties and their dynamic or static interaction. Increasing the friction coefficient was observed to directly impact CP distribution with high localized CP values detected in the locations of the first balloon-vessel interactions (see Figure 12). Another parameter that was observed to highly impact CP values and distribution patterns was diameter ratio (DR): increasing DR from 18% to 40% led to a 4.7-fold increase in CP with a distribution map showing linear patterns that become increasingly pronounced as DR increases. Indeed, a high DR directly affects balloon unfolding and CP pattern considering that the balloon nominal diameter used was larger than the arterial model in all applications (Figure 13). Similarly, an increased vessel thickness and stiffness were found to enhance contact non-uniformity while balloon length showed no significant impact on CP magnitude or distribution pattern. It was then concluded that CP could be identified as both a “driving force” for drug coating transfer to the arterial wall and the intrinsic cause of non uniform drug distribution in coating transfer and that the balloon’s folded configuration and its longitudinal thickness were major contributors to the irregular CP pattern observed.

### 3.3 Computational models of LP

Given the importance of LP on the outcome of PCI, especially when it comes to the use of DCB, it is of interest to study the various aspects to gain a deeper insight into how the process may be improved. For example, a better understanding of the mechanical forces applied, the formation and propagation of cracks in calcified plaques, and the interaction between the various types of LP tools would help clinicians to optimize the procedure. There exists several computational models of angioplasty balloon deployment in arteries e.g., (103–109). However, these models have typically not been developed with lesion modification prior to DCB deployment in mind. While LP is a topic of great interest within the clinical community, it has received significantly less attention in the computational modelling literature.

#### 3.3.1 Angioplasty balloons

Deokar et al. (110) developed a computational model to study the effect of angioplasty balloons during LP in a 3D environment. Particular attention was given to selecting the material model for the atherosclerotic plaque and arterial layers. A third order Ogden function combined with Mullin’s effect was adopted to simulate two kinds of plaque, a soft echo-luminescent and a hard calcified plaque. The artery was modelled using a Holzapfel-Gasser-Ogden (HGO) hyperelastic model with inclusion of arterial softening. Moreover, simulations that included perfect plasticity in the media and plaque were also performed to further investigate stress-strain states (111). Focus was given to calcifications modelled within an echo-luminescent plaque with increasing curvature degree ($90^{\circ }$, $180^{\circ }$, and $270^{\circ }$). A non-compliant balloon was then deployed at 8 atm and Von Mises stresses and strain concentrations were analyzed to determine impact of balloon expansion. Of note was the highly non-uniform stress distribution in the medial layer when compared to the adventitia and the calcified plaque. Around 60%–70% of strain energy was dissipated in the calcified region with increasing damage energy as the plaque degree increased, with lower stresses in the adventitia and media layer in the areas located behind the plaque. It was suggested that this would increase possibility of vascular injury in the non-calcified portions of the plaque.

In another work from Helou et al. (112), angioplasty was modelled to study the effect of balloon type, balloon size and plaque composition on LP outcomes. As in the work of Deokar et al., a 3D cylindrical arterial model was utilized to simulate the artery. However, in this case, the wall was considered as homogeneous with no distinction of arterial layers and the plaque was modelled as a protrusion in the arterial lumen with a material characterisation as lipidic, calcified or mixed (lipid-to-calcified ratio with 30%, 50%, and 70% randomly distributed calcifications). A bilinear hardening isotropic material model was applied in both the arterial layer and the plaque. Semi-compliant and non-compliant angioplasty balloons were taken into consideration at various inflation pressure values. Finally, LP outcomes were tested using stress-strain distribution and Elastic Recoil Ratio (ERR) and Lumen Gain Ratio (LGR) values, and compared to the literature. It was observed that balloon type had no impact on post-procedural outcomes while plaque composition and balloon sizing strongly affected LGR and ERR values. Inflation pressure had a positive non-linear correlation with ERR and LGR, showing that larger balloon sizing for LP treatment determined higher plastic strains. Moreover, it was reported that calcified plaques experienced higher ERR when compared to lipidic ones and it was hypothesized that stiffer plaques experience less compression during balloon expansion when compared to the elastic walls, determining higher elastic recoil values. Differently, LGR was observed to decrease as calcification percentage increased, resulting in higher plastic deformations in more lipidic-like lesions.

#### 3.3.2 Cutting and scoring balloons

Kawase et al. developed a computational model to investigate effectiveness of a scoring balloon in a calcified lesion and clarify the effect of the scoring elements on the plaque (113). The computational simulation was developed to mimic the *in vitro* experimental settings used as a reference for the study, hence a cylindrical 2D arterial cross-sectional geometry was considered. The arterial model was built as a layered cylinder comprising of an external layer of connective tissue, followed by a uniform arterial wall layer and a circumferential calcified plaque. The balloon was designed as a simple thin hollow cylinder with two scoring elements placed $180^{\circ }$ apart, each modelled as a simple circumference. For each element an isotropic linear elastic material was adopted and defined through Young modulus and Poisson’s ratio. The scoring balloon was placed on the luminal portion of the artery and in contact with the initial configuration. Internal pressure was then applied in the balloon up to 18 atm in models with increasing plaque thickness (100–250 μm). The first principal stress was then calculated in the calcified plaque and used as a measure for crack formation as crack propagation was not modelled directly. A comparison of internal pressures values was done between the bench test results and the finite element analysis. For the bench test, the authors calculated the pressure values that initiated crack formation: pressure was applied in the balloon until a visible transmural and longitudinal crack was visible thanks to high speed cameras. This value of pressure was then compared to the internal pressure in the computational analysis, using the first principal stress as an indicator of crack formation. The principal stress was consistently at least three times greater when using the balloon catheter with scoring elements and it concentrated on the exterior of the calcified plaque directly opposite the scoring element.

A different study, developed to evaluate the optimal expansion method for a cutting balloon in a calcified vessel, utilized a similar 2D model for the vessel (114) (cylindrical, artery with respective concentric layers: surrounding tissue, adventitia, media and calcification), albeit the calcification shape was derived from 3D-printed circumferential lesion models. The cutting balloon was modelled based on the Wolverine cutting balloon with elements designed to match the size of the real wolverine cutting blades and placed $120^{\circ }$ apart. Once again, the material model used to define each component of the finite element model was isotropic linear elastic (Young Modulus and Poisson ratio comparable to the work from Kawase et al.). It was observed that the presence of the blades increases the maximum principal stresses, therefore decreasing the pressure value necessary for optimal dilatation. Moreover, to obtain a successful dilatation it is often necessary to apply a series of balloon expansions intermediated by rotations.

**Figure 14:**
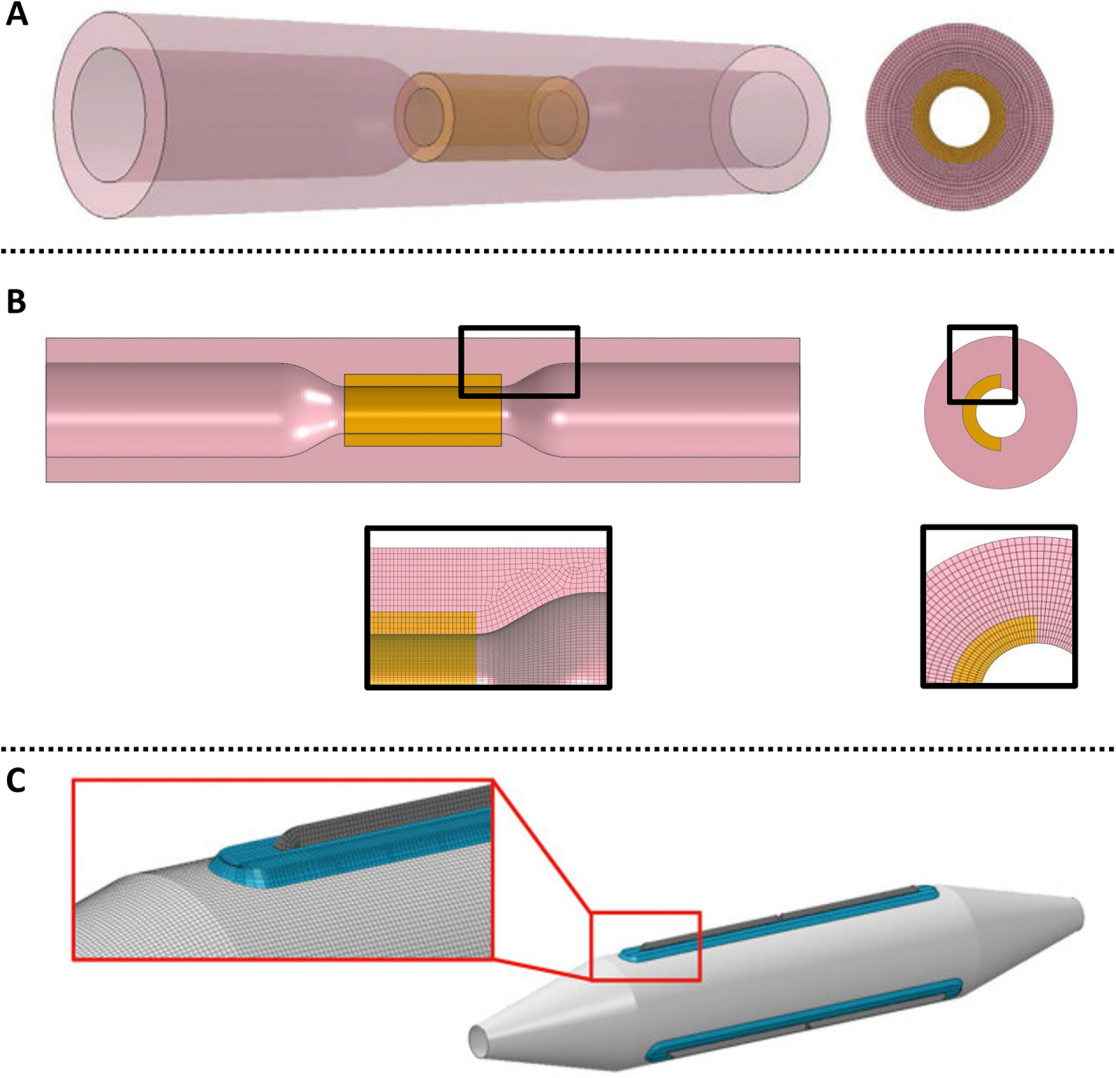
**(A)** 3D coronary model with cylindrical plaque. Obtained from (115), under a Creative Commons [Attribution-NonCommercial-NoDerivatives 4.0 International] license; **(B)** longitudinal cross-section with plaque close ups. Obtained from (116), under the terms of the Creative Commons Attribution License; **(C)** cutting balloon.

The effectiveness of different cutting balloons has also been studied in a series of recent computational models. The primary goals were to determine the appropriate balloon-artery ratio (BAR) in highly calcified lesion through the study of stress levels on the calcification (115) and to assess the impact of balloon expansion technique on procedure outcome (116). The vessel was modelled as a 3D cylinder with an inner rigid shaft to represent the calcified plaque modelled as an elastic isotropic material. In order to study the effect and distribution of stress caused by the blades of the cutting balloon, both a $360^{\circ }$ (Figure 14A) and a $180^{\circ }$ plaque (Figure 14B) were modelled. The balloon was designed with three cutting blades and an isotropic membrane (based on previous computational works) (Figure 14C) (117, 118) and the folding pattern of the balloon was also modelled. As internal pressure is applied, the balloon unfolds and the blades and membrane come in contact with the arterial lumen.

In these computational models, stress and strain were the primary outputs. It has been suggested that tensile stress concentration is related to plaque fracture and vessel dissection at the junctions between healthy and diseased tissue (119, 120). Therefore, the first principal stress was employed to test the stress level in the calcified plaque and the biomechanical interaction between the scoring and cutting balloons and the diseased vessel. It was observed that higher pressure was needed to crack the plaque as the thickness of the plaque was increased and that the efficacy of the blades was higher on thinner plaque portions. The computational analysis demonstrated that stresses were higher in the case of scoring and cutting balloons when compared to plain angioplasty balloons, due to stress concentration in the tissue surrounding the wires and blades. Due to this localized stress concentration being achieved at lower inflation pressure values, modifying balloons can achieve calcified vessels expansion with lower pressure values than plain angioplasty balloons. In the scoring balloon computational analysis (113), the balloon dilatation forces were concentrated around the wire, leading to the formation of cracks on the external portion of the calcified plaque. Cracks were identified using the first principal stress due to calcifications being weak in tensile stress, and compared to experimental data obtained from the bench test settings. The crack propagation behaviour was found to be comparable in both scoring and cutting balloons.

**Figure 15:**
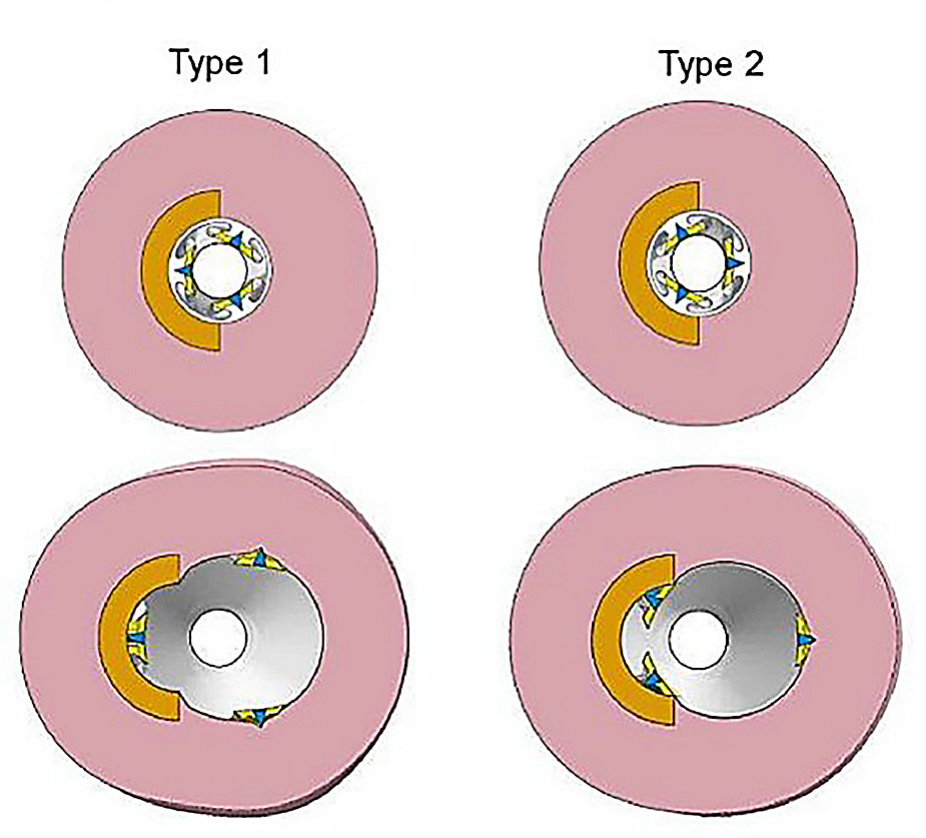
Two models types for balloon expansion: in Type 1 only one blade is placed against the circumferential plaque, in Type 2 two blades are placed against the plaque. Obtained from (116), under the terms of the Creative Commons Attribution License.

In the models that employed a concentric lesion (113–115), it was observed that a BAR of 1:1 for cutting balloons might not be the best choice for clinical practice if the aim is to avoid artery perforation. As a result of the computational analysis, it was suggested that choosing a smaller diameter balloon than the reference artery diameter might be more effective to expand the vessel lumen without the risk of dissection. The study of Zhu et al. evaluated efficacy of cutting balloon technique on an eccentric ($180^{\circ }$) calcified plaque model showing interesting results. In this study, two case scenarios were developed: Type I where one blade was placed against the plaque and Type II where two blades were facing the plaque. It was observed that the Type II case had better outcomes in terms of plaque deformation and expansion than Type I. Also, higher stresses were reached on the plaque using balloons with smaller diameters and lower stresses were observed on the adjacent healthy arterial tissue, meaning that Type II case scenario might not only be more efficient but also safer for LP (Figure 15). It was observed that the spatial configuration of the cutting balloon has direct consequences on the lesion behavior. A two blade configuration was found to induce plaque fracture more easily, therefore reducing vessel dissection. In both simulations, linear elastic material properties were used to simulate balloon, plaque and blades properties.

Song et al. (121) modelled four different scenarios where cutting and angioplasty balloons, with or without a stent, were tested on a calcified coronary artery: (i) non-compliant angioplasty balloon expansion; (ii) cutting balloon expansion; (iii) stent expansion with angioplasty balloon and implantation; (iv) stent expansion with cutting balloon and implantation. The results of the simulations showed that the angioplasty and cutting balloon inflation had better outcomes in terms of plaque fracture than stent implantation followed by the balloons. In fact, the stent was observed to act as a metallic cage that protects the calcified plaques and hinders balloon interaction. Moreover, stent implantation on an intact plaque followed by balloon expansion, either angioplasty or cutting, would require higher pressure levels to break the plaque which could lead to vessel damage or insufficient dilatation. This data supports the importance of LP prior to stent implantation to optimise results.

## 4 Challenges and opportunities in utilising computational models of DCB within clinical practice

DCB have demonstrated promising results in a series of clinical trials, and are now an integral tool for the interventional cardiologist. Despite the progress that has been made, there is still limited understanding of how to optimise their design and use. As with other cardiovascular devices such as DES, computational modelling has the potential to fill this void. However, the computational modelling literature on DCB remains relatively immature. This article has summarized the existing models, mainly focusing on drug delivery from DCB and the importance of LP. Here we discuss how advancements in computational modelling, allied to experimental and clinical testing, can maximise the usefulness of *in silico* models of DCB in the field.

### 4.1 Addressing the limitations of existing computational approaches

Despite the progress that has been made in developing computational models of endovascular devices, the existing literature on DCB has several limitations.

#### 4.1.1 Modelling the balloon

Most studies that report models of DCB use have not included an accurate representation of the balloon. In particular, those studies modelling drug delivery from DCB have incorporated the drug source via a time-dependent flux condition, or a simple coating layer, and have neglected the shape and size of the balloon. As a consequence, no study to date has modelled the simultaneous effects of balloon expansion and tissue deformation on drug delivery, spatial distribution and retention. Notwithstanding, some recent studies have modelled three-dimensional semi-idealized balloon expansion in idealized arteries (84, 103–106), in some cases incorporating the effects of balloon unfolding (102). In addition, the influence of coating microstructure on contact pressure and coating transfer have been investigated (100, 101). While these studies did not directly model the drug delivery, they highlight the potential importance of balloon unfolding, coating microstructure and non-uniform contact pressure on coating transfer and thus drug delivery. It is also worth noting that balloon folding influences drug coating during crimping and drug loss during tracking, aspects which have been explored experimentally, but have yet to be adequately incorporated into computational models.

#### 4.1.2 Increasing the realism of the arterial geometry via imaging

A further limitation of existing models of DCB and LP devices is their reliance on simplified arterial geometries of the target coronary lesion. To our knowledge, only one 3D model has been reported. However, it assumes a single-layer arterial wall with a simplified calcification (97). A range of imaging technologies (e.g., optical coherence tomography (OCT) and IVUS) are now routinely used during PCI to better understand the arterial geometry and lesion morphology. These imaging techniques can be rendered into patient-specific geometries that incorporate disease composition including plaque distribution, lipid burden, fibrous tissue and calcium. Clinical trials have now demonstrated the benefit of this additional information to optimize long-term clinical outcomes (122–125). However, with increasingly complex geometry comes additional challenge. Patient-specific modelling has attracted much interest in the literature, and a number of techniques have been proposed. However, the process of creating a computational geometry from intravascular images needs to be significantly streamlined. Some computational models of DCB have utilised VH-IVUS to create 2D diseased geometries (94–96). These have primarily served the purpose of exploring drug transport and retention, rather than the influence of disease composition on the DCB-artery mechanical interaction. A model that incorporates both complex arterial properties and DCB biomechanics to better understand short and long-term procedural success remains an unmet need.

#### 4.1.3 Developing more realistic and accurate mechanical models of the diseased coronary artery

Hyperelastic (and to a lesser extent, elastic) material models are routinely used to represent the response of arteries to device deployment. When a coronary stent is deployed and left *in situ*, these models can provide good agreement with experimental or imaging data (126). However, predicting the behaviour of diseased vessels following temporary balloon deployment, including DCB, is more challenging. For example most diseased coronary arteries will demonstrate a degree of “recoil” following balloon withdrawal meaning that the lumen reduces in diameter from the point of maximal balloon inflation. The extent of recoil remains challenging to predict but is an important clinical parameter. Somewhat arbitrarily, current DCB guideline statements suggest a residual stenosis of >30% following balloon withdrawal should be regarded as suboptimal and mandate either further LP or stent implantation. Arterial recoil cannot be captured with the use of hyperelastic models, where the lumen would return to the original diameter when the balloon is removed. Thus, inelastic material models of arteries are needed to enhance the accuracy of post-deployment geometry prediction. This is most relevant when complex adjuvant interventions are used prior to DCB deployment

Having an accurate deployment geometry has several important implications, for example, in the simulation of drug transport and retention, arterial fluid dynamics and arterial remodelling. Several studies have explored inelastic arterial models and the mechanical properties of plaque components. Some models were focused on replicating the stress-softening caused by cyclic loading and tension of soft tissue also known as Mullins effect, such as in the work from Balzani et al. (127) where a 2D simplified arterial model was used, or in the work from Conway et al. (128) (focused on stenting), and Maher et al. (129), where a constitutive model was obtained from mechanical testing. In other works, the inelastic properties of the atherosclerotic arteries were modelled taking into consideration plastic deformations either of the plaque (110, 130, 131), of the arterial layers (107, 108, 132, 133) or both (112, 134). Finally, inelasticity has also been interpreted in terms of crack formation and propagation in the works of Versluis et al. (135) and of Gasser et al. (109). However, to the best of the author’s knowledge, these have never been brought together to provide a computational framework able to test device deployment and simultaneous drug delivery, nor subsequent arterial remodelling.

#### 4.1.4 Better estimation of computational model parameters

Computational models of DCB rely on limited literature and often utilize a combination of data from *in vitro*, *ex vivo*, or cell/tissue experiments. The utilization of data from arterial tissue is often derived from various sections of the vasculature, each with its own distinct characteristics. Obtaining data pertaining to diseased human coronary arteries is particularly scarce, but it would be exceedingly valuable for computational models. The task of extending these findings to patient-specific model parameters poses a significant challenge. While mechanical properties can be obtained through an inverse approach using *in vivo* imaging (136–138), drug transport parameters cannot be directly observed or measured. It is imperative to develop methodologies that enable more precise estimation of human mechanical and drug transport parameters.

### 4.2 Unmet clinical challenges

#### 4.2.1 Lesion selection

*De novo* coronary artery stenoses vary considerably in their location within the coronary artery tree, dimensions, severity of stenosis and composition. Our understanding of which lesions may be best suited to DCB treatment remains limited. Randomised clinical trial evidence is restricted to studies of *de novo* lesion in vessels <3 mm (57, 59, 63, 139) with evidence supporting DCB use in larger vessels coming from registry data. Assessment of the lesion is, however, crucial to predicting short and long-term outcome following DCB treatment without stenting. The current generation of industry-leading DES that are low profile, flexible, relatively inert with well-studied drug elution properties and a wealth of long-term clinical follow-up data, represent a high standard of care. Nonetheless, the concept of leaving no residual artificial material behind remains attractive and long-term follow-up data suggests lower rates of MACE with this approach. Algorithms that employ patient and lesion-specific data to support the clinician choice to use a DCB-vs-DES would be of great value.

Early studies of DCB use for ISR have shown that adequate LP improves outcomes (73). In selecting lesions that may be most suitable for DCB treatment, anticipation of the most appropriate LP to achieve angiographic success will be important. Data supports the use of scoring balloons (74), rotational atherectomy (140) and lithotripsy (141) prior to DCB deployment. Adjuvant LP techniques to achieve uniform balloon dilatation to the reference vessel diameter seem vital to ensuring clinical outcomes comparable to DES. Thus models that predict the need for calcium modification techniques according to the lesion morphology and extent of calcification could have significant impact on decision making in the cath lab. As well as the potential for increased risk of complications, there are significant time and cost implications to utilizing these adjuvant devices and this will impact on the cost:benefit of DCB over DES use. Predicting the likely severity and extent of any dissection or arterial recoil following LP will also help limit the need for bailout stenting.

#### 4.2.2 Bifurcation lesions

A particular subset of lesions that demand additional understanding of the utility of DCB use are those involving bifurcations, usually regarded as lesions with a side branch of >2.0 mm in diameter. The most recent European Bifurcation Club guideline document (142) emphasizes a provisional single stent approach with imaging to guide preparation, sizing and deployment. Use of DCB as a default treatment for both main vessel and side branch or as a treatment of the side branch with main vessel DES implantation have both been proposed. Understanding which of these strategies may provide the best short and long-term outcome on a patient- and lesion-specific basis remains limited (143). Modelling of bifurcation intervention has been described to help predict the outcome of DES implantation. A similar approach for DCB use would be of value.

#### 4.2.3 Acute vs. chronic coronary syndromes

The efficacy of DCB use has largely been studied in stable coronary artery lesions including predominantly small vessels and ISR without acute plaque rupture. Acute lesions present a number of features that may increase or decrease the efficacy of DCB. Acute lesion may have considerable thrombus adherent to the wall thus preventing apposition of the DCB surface with the endothelium and potentially reducing transfer of drug. In addition, assessment of vessel size is more challenging in acute lesions, particularly in the context of ST elevation myocardial infarction when flow may be reduced and vasospastic mediators abundant. There is emerging data suggesting that despite these limitations, outcome following DCB use in acute coronary syndromes (ACS) compares with DES implantation. The use of patient factors and imaging to guide the subset of ACS lesions that may be most suited to DCB or DES use has not yet been described.

#### 4.2.4 DCB selection

The portfolio of commercially available, CE marked, DCB has grown significantly in the last years. The majority of balloons continue to have Paclitaxel as the drug but more recently new devices with sirolimus coating have become available. The delivery of sustained amount of sirolimus to the wall remains more challenging and adjuvant technologies such as microvesicles to aid transfer and retention have been proposed. Whether specific drugs and delivery technologies favour particular coronary lesions including bifurcations, calcified lesions or acute lesions with thrombus remains uncertain. Better understanding of which technology may offer the best outcome using modelling techniques to guide clinicians is needed.

The wealth of potential uncertainties makes undertaking adequately powered clinical trials to answer these unmet needs unrealistic. Thus, the prospect of mathematical and statistical *in silico* modelling to improve our understanding of the potential benefits of generic and device specific DCB technology will be important.

### 4.3 Integrating *in silico* modelling within the clinical workflow

The deployment of a DCB requires careful planning and, as things stand, computational models typically only simulate one or two relevant aspects of the overall problem. LP is critical to DCB success (66), yet no published models of DCB have comprehensively addressed this aspect. While some models of particular LP devices have appeared (e.g., scoring/cutting balloons) (113–115), these negelect the drug delivery aspect. Other techniques are less well studied from a structural mechanics point of view, being focused on the fluid dynamics of special devices, e.g., atherectomy and lithotripsy (144–146).

The provision of validated computational models of different LP strategies could help clinical teams to compare and visualise the effect of each therapy prior to use in the patient. There are multiple potential benefits of this approach, including increasing operator confidence, lowering risk of complications and improving procedural efficiency. Moreover, *in silico* simulation can provide an understanding of what might go wrong, enabling the clinical team to be better prepared by ensuring that operator experience and available devices are tailored to the procedure. In addition to facilitating the procedure, *in silico* modelling may also support long-term clinical outcome. Over the subsequent weeks to years following PCI, the artery heals, grows and remodels. If this process occurs in an adverse way leading to further coronary instability, or restenosis then the patient may require a further revascularisation procedure. Currently, further assessment and intervention is driven by patient presentation with recurrent symptoms or an ACS event. There is an opportunity to use *in silico* models of arterial growth and remodelling as part of the assessment of different DCB revascularisation strategies to help predict recurrent presentations. The growth and remodelling literature is advancing in this respect (147–153), but existing models suffer from a series of limitations.

In clinical practice, the concept of pre-procedural planning of PCI procedures has developed considerably in the last decade. The use of intracoronary imaging during PCI has already been highlighted with evidence supporting better clinical outcomes. Algorithms have emerged to support a strategic approach to managing coronary artery calcification (154). The rapid growth of computed tomography coronary angiography has also encouraged the development of applications to assess coronary severity on these images (155–157) and enable pre-procedural planning. These technologies and the supporting clinical trial data demonstrate that computational modelling can inform decision making in the cardiac catheterization laboratory. The potential to further develop *in silico* models to enhance procedural planning and PCI strategies is clear.

## 5 Conclusion

DCB use is set to rapidly increase in the coming years due to the promising results observed in a series of clinical trials and their potential advantages over permanent DES. This article has made the case for computational modelling to play a key role in understanding how these devices may be used optimally in clinical practice. However, a number of technical challenges and opportunities have been identified that must be overcome before the results of computational models may be reliably used in clinical practice.
